# Single atom tungsten doped ultrathin α-Ni(OH)_2_ for enhanced electrocatalytic water oxidation

**DOI:** 10.1038/s41467-019-09845-z

**Published:** 2019-05-14

**Authors:** Junqing Yan, Lingqiao Kong, Yujin Ji, Jai White, Youyong Li, Jing Zhang, Pengfei An, Shengzhong Liu, Shuit-Tong Lee, Tianyi Ma

**Affiliations:** 1 Key Laboratory of Applied Surface and Colloid Chemistry, Ministry of Education; Shaanxi Engineering Lab for Advanced Energy Technology, School of Materials Science and Engineering, Shaanxi Normal University, Xi’an, 710119 People’s Republic of China; 2 Institute of Functional Nano & Soft Materials (FUNSOM), Jiangsu Key Laboratory for Carbon-Based Functional Materials & Devices, Soochow University, Suzhou, Jiangsu 215123 People’s Republic of China; 3 Discipline of Chemistry, School of Environmental and Life Sciences, University of Newcastle, Callaghan, NSW 2308 Australia; 4 Beijing Synchrotron Radiation Facility, Institute of High Energy Physics, Chinese Academy of Sciences, Beijing, 100049 People’s Republic of China; 5 iChEM, Dalian Institute of Chemical Physics, Dalian National Laboratory for Clean Energy, Chinese Academy of Sciences, Dalian, 116023 People’s Republic of China

**Keywords:** Electrocatalysis, Heterogeneous catalysis, Energy

## Abstract

Electrocatalytic water oxidation is a rate-determining step in the water splitting reaction. Here, we report one single atom W^6+^ doped Ni(OH)_2_ nanosheet sample (w-Ni(OH)_2_) with an outstanding oxygen evolution reaction (OER) performance that is, in a 1 M KOH medium, an overpotential of 237 mV is obtained reaching a current density of 10 mA/cm^2^. Moreover, at high current density of 80 mA/cm^2^, the overpotential value is 267 mV. The corresponding Tafel slope is measured to be 33 mV/dec. The d^0^ W^6+^ atom with a low spin-state has more outermost vacant orbitals, resulting in more water and OH^−^ groups being adsorbed on the exposed W sites of the Ni(OH)_2_ nanosheet. Density functional theory (DFT) calculations confirm that the O radical and O-O coupling are both generated at the same site of W^6+^. This work demonstrates that W^6+^ doping can promote the electrocatalytic water oxidation activity of Ni(OH)_2_ with the highest performance.

## Abstract (web summary)

Electrocatalytic water splitting for hydrogen and oxygen generation provides an attractive path to obtain clean energy, but the half reaction of oxygen evolution remains the bottleneck for the progress. Here, the authors show single atom tungsten doped ultrathin α-Ni(OH)_2_ exhibits enhanced performance in electrocatalytic water oxidation.

## Introduction

Electrocatalytic water splitting for hydrogen and oxygen generation provides an attractive path to obtain clean energy via the conversion and storage of intermittent solar and wind energies; however, the half reaction of oxygen evolution remains the bottleneck for such progress^1–5^. The oxygen evolution reaction (OER) is kinetically sluggish and needs the necessary two steps of O–H bond breaking and attendant O–O bond formation, which includes the transfer of four electrons^4–19^. The design of a considerable OER electrocatalyst is needed. In this case, the noble metal-based catalysts, for example IrO_2_ and RuO_2_, exhibit attractive OER activity, but their rare nature hinders their large-scale application. A great many non-noble metal-based alternative OER catalysts using abundant 3d metals (Fe, Co, Mn, and Ni) have been studied with increasing activity and stability in recent years^9,10,20–26^. In particular, Ni(OH)_2_-based samples have garnered a great interest with studies showing their encouraging water oxidation potential^4,9^. In addition, the doping of elemental Fe with Ni(OH)_2_ at low content levels has been shown to greatly increase the OER performance^27^. However, substantial progress in the development of Ni(OH)_2_-based OER catalysts with enhanced activity is still needed.

For the Fe-doped Ni(OH)_2_ sample, the detailed OER pathways have been shown through several manners of occurrence. In the preliminary works, Fe doping is considered to improve the electrical conductivity^27^ and change the electronic structure, anodically shifting the Ni redox potential^28,29^. Boettcher confirmed that Fe exerted a partial-charge transfer activation effect and then promoted the OER of the Ni sample^30^. By using operando X-ray absorption spectroscopy and computational methods, Bell et al. proposed that the Fe doping in NiOOH produced short Fe–O bonds. These active bond sites were said to have resulted in the high OER performance with low overpotentials and optimal adsorption energies of OER intermediates being observed^31^. Stahl et al. first detected the formation of Fe^4+^ during the OER process by Mössbauer Spectroscopy and reported that the species was not kinetically competent to serve as the active site in water oxidation^32^. Strasser concluded that Fe could suppress the oxidation process of Ni species from + 2 to + 3/ + 4 and help the lower-valent states of the Ni centers have a higher OER activity^33^. The Fe sites in Ni-based hydroxides are almost accepted to be the critical component of the record activity of water oxidation. To further confirm that reactive Fe sites were responsible for the exceptional OER, the introduction of Fe into the NiO_x_H_y_ from solution was performed with the unaltered conclusion that its precise role remains unclear^34^. Although the replacement of Fe^3+^ to Ni^2+^ in the Ni(OH)_2_ sample has been shown to enhance oxygen evolution through extensive study, the core part of the water oxidation on the surface of the Ni(OH)_2_-based sample, that is, the reaction behavior on the exposed/contacted Ni(OH)_2_ surface is rarely studied and reported^28,34–37^. Moreover, the OER performance of Ni(OH)_2_-based electrocatalysts still needs improvement by doping, and the finding of a suitable element remains to be developed.

On the electrocatalytic process, there is an electric double layer (Helmholtz layer, HL) at the catalyst|solution interface as shown in Supplementary Fig. 1. The electrical circuit is completed through three resistance measures i.e., the catalyst, catalyst|solution interface, and the solution. The HL is important and determinative for the final electrocatalytic efficiency owing to the induced capacitive and resistive characteristics resultant from the changing of a solid to solution^38–40^. In the case of water splitting, the adsorption and desorption behaviors of H_2_O molecules and OH^−^ groups in the HL will directly influence the hydrogen or oxygen generation^41,42^. Therefore, the specifics of the coordination of the exposed elements (such as Ni or Fe) should be studied further to potentially improve the performance^35^. The appropriate chemical environment, the strength of the bond between the electrode and intermediates and the electron migration resistance are the key factors in leading to a significant OER enhancement^43^. Recently, Shin et al. found that both Ni^4+^ and Fe^4+^ played essential roles in the OER. The high spin d^4^ Fe^4+^ site was favorable for stable O· radical formation with the low spin Ni^4+^ more favorable for O–O bond coupling^44^. In addition, Zhang et al. reported that the W doping can provide the near-optimal adsorption energies for OER intermediates in FeCo oxy-hydroxide samples^14^, and single-atom doping has been reported to be useful for assisting suitable sites for intermediates formation^45–48^. It is still then necessary to find one suitable reaction site for achieving both the formation and migration of intermediates.

Herein, we report that W^6+^ doping can boost the OER performance of Ni(OH)_2_ greatly. Owing to the d^0^ characteristic, the W^6+^ doping sites exhibit exothermic H_2_O adsorption and O radical formation reactions, differing from the endothermic reactions otherwise reported. Furthermore, the formation of the O radical and O–O coupling both happen at the W^6+^ sites. In conjunction, the W-doped sample exhibits low reaction potentials at high current densities.

## Results

### Insight into the water oxidation on Ni(OH)_2_

**Fig. 1:**
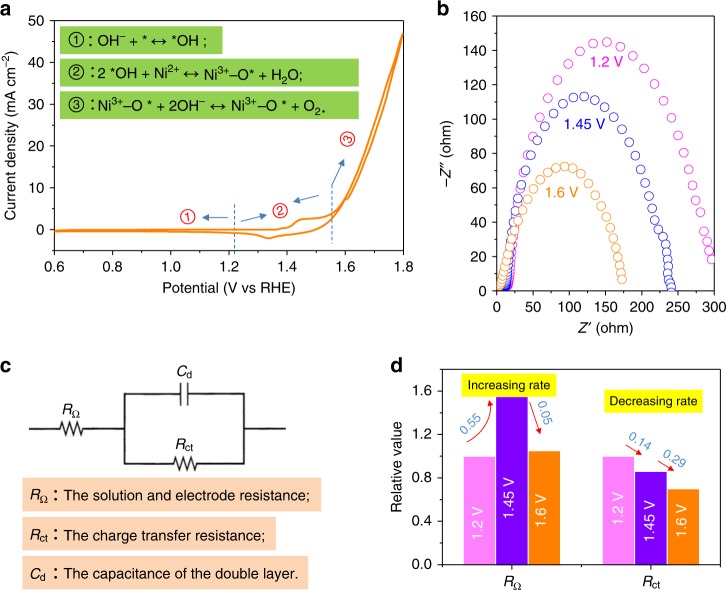
Electrochemical analysis of Ni(OH)_2_ for OER processes. **a** Cyclic voltammetry (CV) for Ni(OH)_2_ at the potential range of 0.6–1.8 V; **b** Nyquist plots of Ni(OH)_2_ under the three potentials of 1.2, 1.45, and 1.6 V. **c** Equivalent electric circuit for the EIS plots fitting. **d** The change rate of the fitted resistance values in Supplementary Table 1. The corresponding increasing and decreasing rates are for R_Ω_ and R_ct_, respectively. The CV and EIS were measured in a 1 M KOH solution. Note that the proposed reaction pathways in (**a**) are not chemically conserved

At first, the electrocatalytic processes of Ni(OH)_2_ were studied to check the interfacial effect on the OER. On the electrocatalytic water oxidation by Ni(OH)_2_, one obvious process attributable to the Ni^2+^ ↔ Ni^3/4+^ peak (*ca*. 1.45 V in Fig. 1a) in the cyclic voltammetry plots was detected^30–37^. This process happened together with the formation of the O*, which is an essential intermediate (which couples with one OH^−^ group to generate *OOH) for oxygen evolution^44^. Figure 1a gives the CV curve of Ni(OH)_2_, and the inset shows the corresponding oxygen evolution intermediate pathways: at the low potential (region ➀), *OH generation; in the mid potential (region ➁), the above-mentioned process occurs; and then the O_2_ will generate under high potential (region ➂). The three simplified processes of water oxidation involve the HL and include *OH species adsorption, *O radical formation, and *OOH transformation and desorption^45^. To detect the interfacial resistance of the corresponding process, three potentials of 1.2, 1.45, and 1.6 V were applied for the electrochemical impedance spectroscopy (EIS) measurement as shown in Fig. 1b. Supplementary Table 1 shows the fitted values based on the equivalent electric circuit (Fig. 1c). Clearly, R_Ω_, the resistance of the solution and electrode, presented a slightly increasing trend from 10.8 to 17.5 Ω/cm^2^ under the potential of 1.2 to 1.6 V; meanwhile, the R_ct,_ the charge transfer resistance, gave a decreasing trend from 277.4 to 168.6 Ω/cm^2^. The above increasing and decreasing trends suggested the change (Ni^2+^ → Ni^3/4+^) of the electrode surface. To obtain further information, the change rate of fitted resistance values were carried out as shown in Fig. 1d. The increasing and decreasing rates of 1.45 V compared with 1.2 V are 0.55 and 0.14, respectively, meanwhile, the rates for the 1.6 V condition compared with 1.45 V are 0.05 and 0.29, respectively. The slight change confirms that the process of Ni^2+^ → Ni^3/4+^ at the lower potential is the decisive step for the overall water oxidation. Based on the above experimental results and analysis, an excellent OER Ni(OH)_2_-based catalyst should have the suitable outermost orbits for *OH species adsorption and for stabilization of the *O radical^49,50^. Moreover, if it has the preferred O–O coupling ability, then high water oxidation efficiency will be expected.

**Fig. 2:**
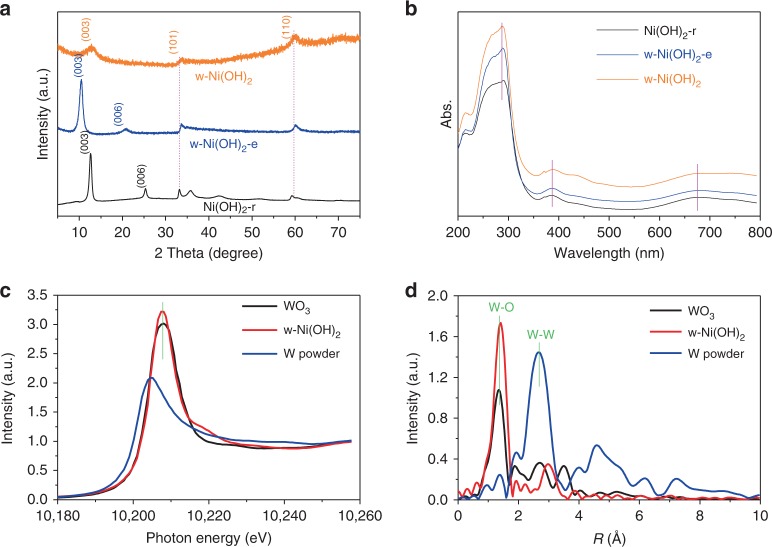
Physicochemical property characterization. **a** XRD patterns of the reference Ni(OH)_2_-r, w-Ni(OH)_2_-e, and w-Ni(OH)_2_ samples; **b** UV–Vis absorption spectrum; **c** The W K-edge XANES spectra, and **d** the corresponding K^2^-weighted Fourier transform spectra of w-Ni(OH)_2_, WO_3_, and W powder were used as the reference samples

Synthesis of W single-atom doped Ni(OH)_2_. Bare Ni(OH)_2_ and doped samples were synthesized through the alcohothermal method using an NiCl_2_–ethanol and WCl_6_–ethanol solution (Experimental section and Supplementary Fig. 2). Figure 2a shows the XRD patterns of the obtained samples. For bare Ni(OH)_2_, (Ni(OH)_2_-r), all peaks were assigned to the α-Ni(OH)_2_ phase (PDF: 38-0715). The tungsten-doped sample (w-Ni(OH)_2_-e) also gave the typical α-Ni(OH)_2_ XRD signals with the obvious difference: the peaks of (101) and (110) loaded at 2 theta of 33.4 and 59.9 degree maintained their location, but the peaks of (003) and (006) showed a left shift compared with the Ni(OH)_2_-r sample. As we know, for the layered Ni(OH)_2_ sample, the shift of the (003) peak to the low diffraction angle suggested that the interlayer spacing between the signal from the octahedral Ni(OH)_6_ layer had been broadened. The W element has a more unoccupied outermost electron orbital than Ni, and the replacement of one Ni by one W in the layered Ni(OH)_2_ structure was expected to generate some additional adsorption sites on W. In our present work, the ethanol or other relative groups would adsorb on the exposed W sites and then widen the layered structure. When the w-Ni(OH)_2_-e sample was treated by ultrasound in the ethanol solution, the final w-Ni(OH)_2_ sample was obtained. As shown in Fig. 2a, only three XRD peaks with low intensity were detected, and the (003) peak exhibited a right shift when compared with the w-Ni(OH)_2_-e sample. The results confirmed that the ethanol exfoliation is serviceable for obtaining the thin layered W-doped Ni(OH)_2_ sample. We also tested the effect of ultrasound time on the final layered result (Supplementary Fig. 3). Supplementary Fig. 4a gives the FTIR spectra of the studied samples. Three peaks relative to the OH group loaded at 484, 1631, and 3440 cm^−1^ were detected^51,52^. Supplementary Fig. 4b shows the Raman spectra of the samples. The relative peaks for the three main OH groups at 1283, 2930, and 3645 cm^−1^ can be found with a decreasing intensity from Ni(OH)_2_-r to w-Ni(OH)_2_^51^. Supplementary Fig. 5 shows the X-ray photoelectron spectroscopy (XPS) results. No obvious difference can be detected^53^.

Figure 2b shows the typical UV–Vis absorption spectrum. No obvious main peak shifts or WO_3_-based signals could be detected, confirming that the W doping did not change the electronic structure of the Ni(OH)_2_ sample. Moreover, the above UV–Vis spectra results suggested the low W content doping or lattice doping, which was thought to have occurred. To further confirm this, the UV–Vis signals of WO_3_, 1%, 2 and 3% WO_3_ loaded Ni(OH)_2_ samples were tested as shown in Supplementary Fig. 6. Obviously, the WO_3_-loaded Ni(OH)_2_ samples show the red-shift optical absorption even at a low content (1%), suggesting that W doping of the w-Ni(OH)_2_ sample was indeed lattice doping via single- or multi-atomic doping. The X-ray absorption spectroscopy (XAS) measurements were carried out to further confirm the atomic doping of W element. WO_3_ and W powder were used as the reference samples. As shown in Fig. 2c, the normalized X-ray absorption near-edge structure (XANES) spectra of W K-edge, the intensity of prominent peak of w-Ni(OH)_2_ or WO_3_ is higher than W powder, suggesting the oxidized electronic structure of W element in w-Ni(OH)_2_. Moreover, the position of the prominent peak of w-Ni(OH)_2_ was close to that of WO_3_ (10208.07 eV), revealing the dominance of W^6+^ in w-Ni(OH)_2_. The above observation coincides well with the XPS results (Supplementary Fig. 5). Further structural information about W atoms can be obtained from the extended X-ray absorption fine structure (EXAFS). The K^2^-weighted Fourier transform spectra of w-Ni(OH)_2_ and the reference samples in R space are shown in Fig. 2d. Typically, the EXAFS curve of W power shows a dominant peak at 2.6 Å, corresponding to the W–W coordination. In clear contrast with this common observation, the sample of w-Ni(OH)_2_ and WO_3_ only show the one peak located at ca. 1.3 Å, which can be assigned to W–O coordination. Importantly, in comparison with WO_3_ (1.31 Å), some appreciable shift of W–O peak (1.39 Å) for w-Ni(OH)_2_ can be detected, confirming the different W–O environment. Supplementary Fig. 7 shows the corresponding atomic force microscope (AFM) images of the studied samples, in which Ni(OH)_2_-r, w-Ni(OH)_2_-e, and w-Ni(OH)_2_ show the thickness of 8, 12, and 8 nm, respectively.

**Fig. 3:**
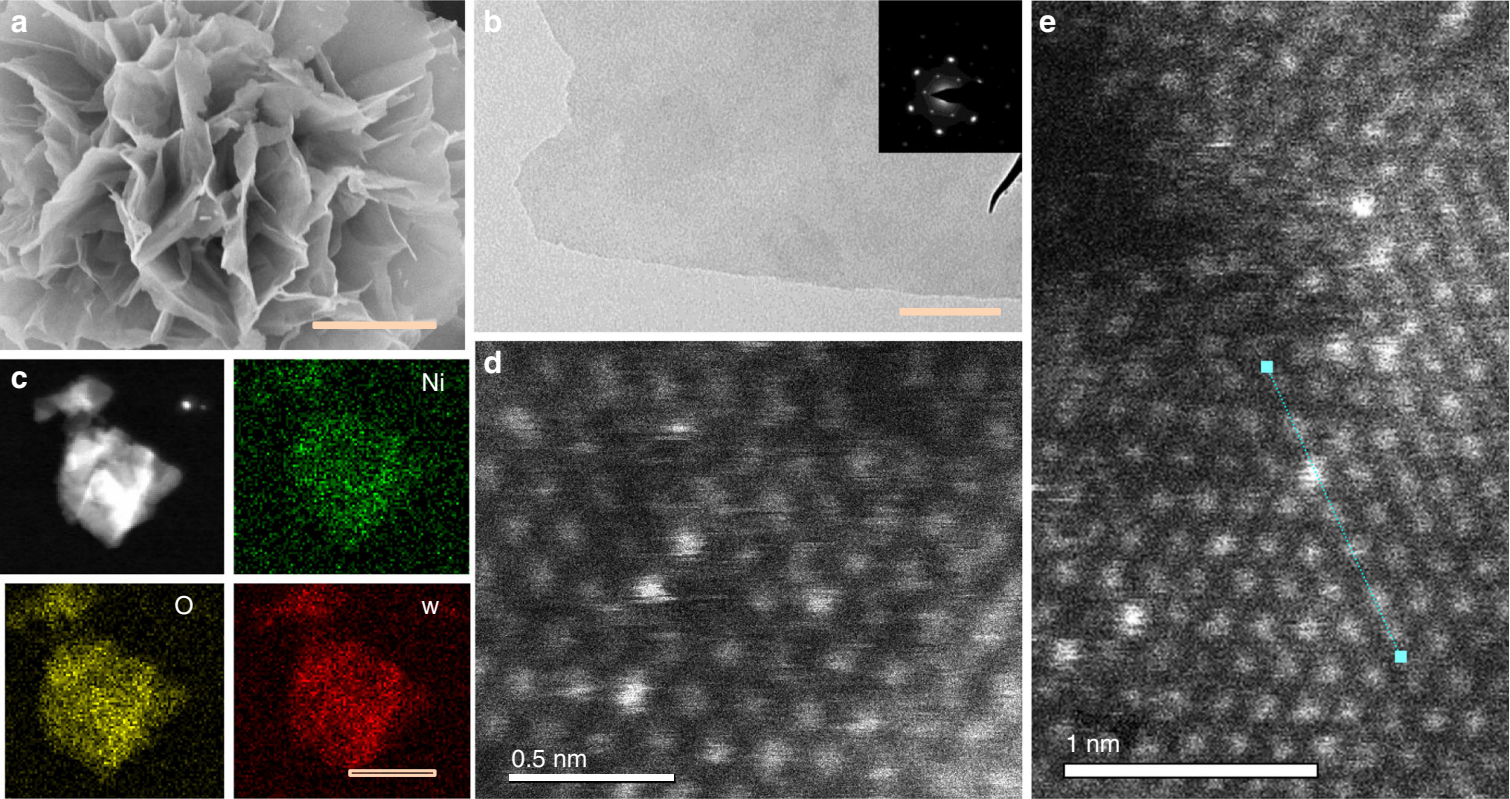
Electron microscope analysis of the w-Ni(OH)_2_ sample. **a** SEM image, scale bar: 100 nm. **b** TEM, the inset shows the corresponding electronic diffraction spectra, scale bar: 100 nm. **c** The HADDF result and the corresponding element mapping, scale bar: 100 nm. **d, e** HAADF-STEM image, the corresponding element contrast result in **e** can be found in Supplementary Fig. 9d, the scale bars are 0.5 and 1 nm for **d** and **e**, respectively

Figure 3 gives the electron microscope analysis of the w-Ni(OH)_2_ sample. According to the SEM image (Fig. 3a), the layered structure of w-Ni(OH)_2_ was found to be clearly evident. The reference samples i.e., Ni(OH)_2_-r and w-Ni(OH)_2_-e also gave the typical layered image (Supplementary Fig. 8). Figure 3b shows the corresponding TEM result of the w-Ni(OH)_2_ sample, the thin layered structure was here further confirmed. The inset shows the typical electronic diffraction spots with the hexagonal structure of α-Ni(OH)_2_ clearly present. The TEM images of reference samples can be found in Supplementary Fig. 9, Fig. 3c gives the HADDF image and the corresponding element mapping. Clearly, the Ni(OH)_2_ structure was doped by the W element uniformly. To directly find the W in the Ni(OH)_2_ lattice, HAADF-STEM was further carried out. As shown in Fig. 3d, the light spots originated from the W element, which had been doped into the lattice and did not change the elements hexahedral arrangement (Fig. 3e). Supplementary Fig. 10a shows the local enlarged HADDF–STEM image and the corresponding mode of the signal by the W-doped Ni(OH)_2_ hexagon structural unit, clearly suggesting that the signal by W doping has been obtained based on our present synthesized process. Moreover, according to the model of the atomically doped W atoms in the Ni(OH)_2_ structure in Supplementary Fig. 10b, the above XAS results confirm the atomic doping of W atoms into Ni(OH)_2_. Supplementary Fig. 11 gives the contrastive HADDF–STEM images of pristine and doped Ni(OH)_2_ samples. Clearly, the W-doped sample gives many disordered sites in comparison with the typical symmetric hexagon structure of the pristine Ni(OH)_2_ sample, further exemplifying that the W doping can change the lattice structure of Ni(OH)_2_. For determining the mass ratio of W to Ni, Inductively Coupled Plasma-Mass Spectrometry (ICP–MS, aurora M90) was used. The atomic ratio was detected to be 2.985: 99.55, near the theoretical value (Supplementary Table 2).

### Electrocatalytic water oxidation performance

**Fig. 4:**
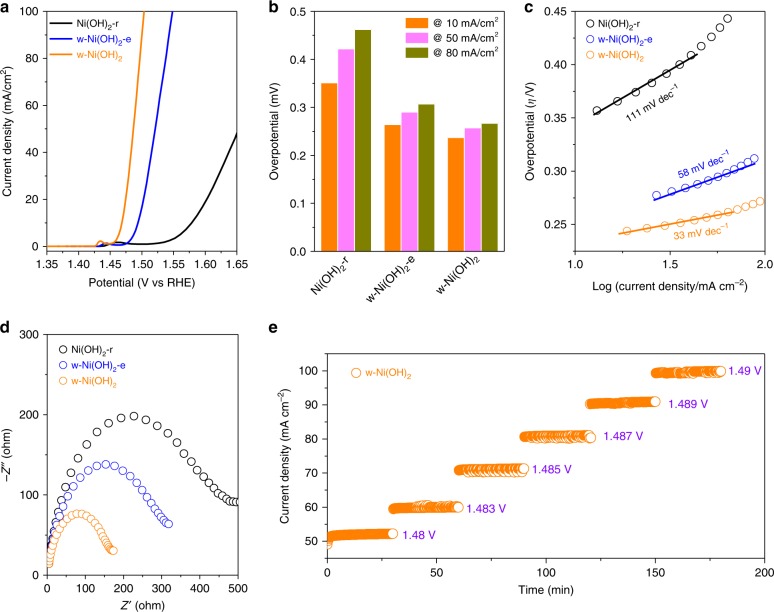
Electrocatalytic water oxidation performance. **a** 95% iR-corrected polarization curves of reference Ni(OH)_2_-r, w-Ni(OH)_2_-e, and w-Ni(OH)_2_ samples. **b** The overpotentials required for j = 10, 50, 80 mA cm^−2^ with different electrodes. **c** Tafel plots for Ni(OH)_2_-r, w-Ni(OH)_2_-e, and w-Ni(OH)_2_ electrodes. **d** EIS Nyquist plots of the Ni(OH)_2_-r, w-Ni(OH)_2_-e, and w-Ni(OH)_2_ electrodes under the bias potential of 1.48 V. **e** Chronopotentiometric measurements of OER at various current densities using w-Ni(OH)_2_ as a catalyst. All the tests were carried out in 1 M KOH solution (O_2_-saturated)

The electrocatalytic activity of the Ni(OH)_2_-r, w-Ni(OH)_2_-e, and w-Ni(OH)_2_ samples toward OER in a 1 M KOH aqueous solution was measured as shown in Fig. 4. As expected, the polarization curve of the w-Ni(OH)_2_ sample gave the best catalytic activity among the three electrodes, delivering a much higher current density than the others at the same overpotential (Fig. 4a). Typically, all the Ni(OH)_2_ samples showed the obvious Ni^2+^ ↔ Ni^3/4+^ peak at the potential of *ca*. 1.45 V. Furthering the anodic potential, the control material pure Ni(OH)_2_-r sample presented a slowly climbing line, indicating a gradual increase in current density. This was in contrast to the W-doped sample which showed a significantly steeper response at a lower potential. We next compared the overpotentials at three catalytic current density values of 10, 50, and 80 mA/cm^2^ as shown in Fig. 4b. The w-Ni(OH)_2_ electrode required an overpotential of 237 mV for reaching the current density of 10 mA/cm^2^, where the reference samples of Ni(OH)_2_-r and w-Ni(OH)_2_-e required 351 and 264 mV, respectively. Note that the potential value of the control Ni(OH)_2_-r sample at the current density of 10 mA/cm^2^ was 351 mV, which is lower than those reported^15,17–19^. At a higher current density i.e., 80 mA/cm^2^, the two samples of w-Ni(OH)_2_-e and w-Ni(OH)_2_ gave overpotential values of 290 and 257 mV, respectively. Please note that the W-doped thin layered Ni(OH)_2_ i.e., w-Ni(OH)_2_ showed the lowest overpotential at current densities of 50 and 80 mA/cm^2^ amongst reported literature values of Ni(OH)_2_-based OER catalysts (Supplementary Table 3). The kinetic parameters of the studied electrodes were further calculated from the corresponding polarization curves by plotting overpotential against log (*j*). As shown in Fig. 4c, Ni(OH)_2_-r, w-Ni(OH)_2_-e, and w-Ni(OH)_2_ showed Tafel slopes of 111, 58, and 33 mV/dec, respectively. To get a direct comparison, commercial RuO_2_, a commonly used electrocatalyst for OER, was prepared and tested as shown in Supplementary Fig. 12, the overpotentials recorded were 350 and 470 mV at the current density values of 10 and 50 mA/cm^2^, respectively. Moreover, the RuO_2_ electrode gave a Tafel slope of 92 mV/dec.

It was thought that the higher intrinsic catalytic activity of w-Ni(OH)_2_ may have been due to its higher conductivity over w-Ni(OH)_2_-e or Ni(OH)_2_-r. To confirm this hypothesis, EIS plots of the three samples were carried out as given in Fig. 4d. According to the above detailed analysis of the EIS measurements (Fig. 1), the bias voltage was set to be 1.48 V. Clearly, the sample of Ni(OH)_2_-r gave the biggest circular arc, followed by w-Ni(OH)_2_-e and then w-Ni(OH)_2_, suggesting that the W element doping improved the electrical conductivity which was then further improved by the exfoliation strategy. Supplementary Table 4 gives the corresponding fitted results based on the same equivalent electric circuit in Fig. 1c. Clearly, the R_ct_ values show the rapid decrease from Ni(OH)_2_-r to w-Ni(OH)_2_, further confirming that the thin-layered W-doped sample had a relatively higher carrier mobility across the HL i.e., the catalyst|solution interface. Furthermore, chronopotentiometry experiments with multiple current steps were carried out to examine the rapid response of the carrier migration as shown in Fig. 4e. Attention was only paid to the relative high current density, which was increased from 50 to 100 mA/cm^2^. The response change of applied potentials was answered by corresponding stable current values consistently, confirming the excellent carrier migration across the interface of the electrode and solution. Supplementary Fig. 13a gives the comparison of the activity between the initial surface and of the surface after 1000 cycles, where no obvious change was detected. The XRD result also exhibited similar patterns (Supplementary Fig. 13b), and indistinguishable SEM images in Supplementary Fig. 13c of the samples can further be found for additional confirmation of structural similarity before and after 1000 cycles. Furthermore, XPS was carried out to confirm the stability of the w-Ni(OH)_2_ structure. As shown in Supplementary Fig. 14, no obvious change in the wide survey spectra was found. Moreover, the W 4 f XPS spectra of the initial, after 10 h sonication and after long-time stability test was also unchanged. To further confirm the stability of the surface W atoms in the Ni(OH)_2_ lattice, the concentration of W in the solutions under the different reaction time at 30 mA/cm^2^ was studied using ICP–MS. As shown in Supplementary Fig. 15, the W signals almost keep the same, suggesting its remarkable stability. The corresponding element mapping and the STEM images have also been given as shown in Supplementary Figs 16 and 17. No obvious change could be detected.

We also tested the relationship between elemental W content in the Ni(OH)_2_ and the final OER activity. According to the polarization curves in Supplementary Fig. 18, the W content played an essential role for the water oxidation in this study and it was concluded that the 3% W content (atomic ratio, W/Ni) was the optimal doping level. It was also shown that an increase or decrease of as little as 1% W dopant will decrease the catalytic performances (Supplementary Fig. 18a). The corresponding Tafel slopes in Supplementary Fig. 18b clearly show the change. Furthermore, we observed that the pH of the solution used for water oxidation was also crucial. The four pH solutions i.e., pH = 13.6, 12, 11, and 10 were prepared, and Supplementary Fig. 19 describes the resultant OER characteristics. The water oxidation activity decreased sharply from high-to-low pH solution, suggesting that the concentration of OH^−^ groups around the electrode surface was very important for OER.

More analysis is needed to clarify the reaction sites of the single-atom W-doped material, w-Ni(OH)_2_. According to Fig. 4a, the oxidation peaks of Ni^2+^ to Ni^3/4+^ at ca. 1.45 V show a slight positive shift for the W-doped samples. These results suggest that single-atom W doping can promote carrier migration across the catalyst|solution interface and weaken the oxidation step of Ni^2+^. The EIS plots (Fig. 4d) support this as the charge transfer resistance, R_ct_ is significantly lower for w-Ni(OH)_2_, see Table S4. The in situ Raman spectra of pristine and W-doped Ni(OH)_2_ was carried out to further verify this. As shown in Supplementary Fig. 20, the Raman shift peaks at *ca*. 440 and 493 cm^−1^ can be assigned to Ni(OH)_2_ signals, meanwhile, the peaks at ca. 477 and 550 cm^−1^ belong to the NiOOH^18^. It was deduced that the Ni(OH)_2_ was being oxidized to NiOOH, and the W-doped sample retained part of the Ni(OH)_2_ phase. This directly correlates with our previous assertion, that the doped W can assist in the migration of carriers from the catalyst to solution and weaken the oxidation effect of the electrocatalyst Ni(OH)_2_.

To better understand the effect of surface W sites on migrating carriers, the OER performance of the reference sample, i.e., WO_3_/Ni(OH)_2_ was also carried out. The STEM image (Supplementary Fig. 21a) clearly showed that the WO_3_ nanoparticles were only loaded on the Ni(OH)_2_ surface, not dispersed through it. Although the OER activity was a little enhanced by WO_3_ loading (Supplementary Fig. 21b), the oxidation potential of the peak of Ni^2+^ to Ni^3/4+^ was almost the same. This indirectly suggests that the W doping could weaken the carriers stay on Ni(OH)_2_. To further verify the advantage of our nanosheet sample, we also synthesized a nanoparticles sample of pure Ni(OH)_2_. As shown in Supplementary Fig. 22, the nanosheet sample shows a better OER performance at high current density than the reference nanoparticle material. We also tried other element doping using Nb, Mo, and Ta, and the corresponding OER performance was shown in Supplementary Fig. 23. The doped samples all exhibited enhanced activity compared with the pure Ni(OH)_2_ sample. However, their improved performance is limited in comparison with W element doping, suggesting that its unique electronic structure may be the reason for the enhanced performance. The specific activity of the samples is also an important parameter to suggest the intrinsic activity. The unit specific surface area values of the three catalysts (Ni(OH)_2_-r, w-Ni(OH)_2_-e, and w-Ni(OH)_2_) were tested to be 45, 46, and 58 m^2^/g, respectively. Supplementary Fig. 24 shows the corresponding specific activity. Clearly, the w-Ni(OH)_2_ sample still gives the best performance.

To obtain more information regarding the water oxidation process, an electrochemically active surface area (ECSA) investigation of w-Ni(OH)_2_ was carried out under the three pH values (pH = 13.6, 11, and 10). The characteristic CV curves of w-Ni(OH)_2_ with different scan rates are shown in Supplementary Fig. 25a–c. By plotting the ∆*j* at a certain potential against the scan rate, a linear slope that is twice the double-layer capacitance (C_dl_) can be obtained, and the C_dl_ can be used to determine the corresponding ECSA^18,19,53^. For our experiment, we used a potential of 1.45 V (vs. RHE) to obtain the ECSA, the results of which are given in Supplementary Fig. 23d. The slopes at pH = 13.6, 11, and 10 are 0.01, 0.0058, and 0.0042 mF cm^−2^, respectively. This suggested that the C_dl_ at a high pH was responsible for a more electroactive surface. EIS plots of the w-Ni(OH)_2_ sample under the three different pH conditions were further carried out. The corresponding Nyquist plot of the pH = 13.6 solution displayed the smallest semicircular arc (Supplementary Fig. 26), followed by pH = 11 and 10, suggesting that the resistance of the HL here in a high-pH solution will return a relatively small impedance value. Moreover, the fast carriers adsorption–desorption and migration properties in the HL could also be obtained from the EIS result. To get the detailed information about the W element promoting the carriers migration in the HL of Ni(OH)_2_, CV curves of the reference Ni(OH)_2_-r and bulk w-Ni(OH)_2_-e samples were carried out under a pH of 13.6. As shown in Supplementary Fig. 27a and b, both of them gave typical closed curves under the different scan rates. We further calculated the C_dl_, as shown in Supplementary Fig. 27c. The samples of Ni(OH)_2_-r and w-Ni(OH)_2_-e gave the slopes of 0.006 and 0.002, both of them were smaller than that of the w-Ni(OH)_2_ sample. This clearly suggested that the efficient property of the W-doped sample was the boosting of carrier migration across the electrode|solution interface. The turnover frequency (TOF) of Ni(OH)_2_-r and w-Ni(OH)_2_ were calculated to be 0.006 and 0.74 s^−1^, respectively. The W-doped sample shows a TOF 100 times higher than the pristine Ni(OH)_2_, seen in Supplementary Table 3, further confirming that the W doping can boost the OER activity. We also tried the practical water-splitting devices of the two-cell electrolyzer as shown in Supplementary Fig. 28. The corresponding OER performance shows the similar curves in the LSV plots, but a little high current response in the high potentials.

### W-doped Ni(OH)_2_ OER mechanism

**Fig. 5:**
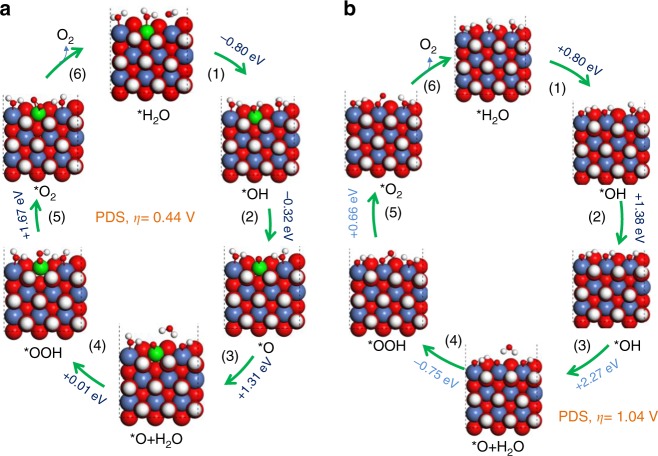
DFT calculation. Mechanism of OER on (**a**) W-doped Ni(OH)_2_ and (**b**) bare Ni(OH)_2_catalyst based on DFT calculations, in which red, white, green, and blue atoms represent the oxygen, hydrogen, nickel, and tungsten atoms, respectively. Note that the symbols of “ + ” and “−” represent endothermic and exothermic free energy. For the step from State 6 to State 1, 1.0 eV, and 0.4 eV endothermic free energy values were required for the doped and bare samples, respectively, where these free energy changes are not shown above

After the experimental characterization, density functional theory (DFT) calculations were implemented to unfold the microscopic mechanism of OER enhancement on W-doped Ni(OH)_2_. The six main sequential steps of water oxidation were explored:

1 $${\mathrm{H}}_{\mathrm{2}}{\mathrm{O {\hskip 1pt} + {\hskip 1pt} }} \ast \to \ast {\mathrm{H}}_{\mathrm{2}}{\mathrm{O}}$$

2 $$\ast {\mathrm{H}}_{\mathrm{2}}{\mathrm{O}} \to \ast {\mathrm{HO + H}}^{\mathrm{ + }}+{\mathrm{e}}^{\mathrm{ - }}$$

3 $$\ast {\mathrm{HO}} \to \ast {\mathrm{O + H}}^{\mathrm{ + }} + {\mathrm{e}}^{\mathrm{ - }}$$

4 $$\ast {\mathrm{O + H}}_{\mathrm{2}}{\mathrm{O}} \to \ast {\mathrm{OOH}}_{\mathrm{2}}$$

5 $$\ast {\mathrm{OOH}}_{\mathrm{2}} \to \ast {\mathrm{OOH + H}}^{\mathrm{ + }} + {\mathrm{e}}^{\mathrm{ - }}$$

6 $$\ast {\mathrm{OOH}} \to \ast {\mathrm{ + O}}_{\mathrm{2}}{\mathrm{ + H}}^{\mathrm{ + }} + {\mathrm{e}}^{\mathrm{ - }}$$

where * represents the element of the surface reaction sites. The OER computational model (shown in Supplementary Fig. 29) adopted two layered α-Ni(OH)_2_ intercalated with Cl^−^ and H_2_O molecules and then an edge Ni atom was substituted by a W atom. Complete OER processes on the W-doped Ni(OH)_2_ and Ni(OH)_2_ were shown in Fig. 5, in which the six reaction steps involved four electron–proton combinations^43^. In the case of w-Ni(OH)_2_ (Fig. 5a), one water molecule was adsorbed on the exposed W^6+^ site (State 1), and then proceeded through two deprotonation steps (State 2 and State 3) releasing energies of 0.76 eV and 0.34 eV, respectively. The adsorbed O radical then attracted one water molecule (State 4) and required 1.31 eV reaction free energy to proceed. After that, the adsorbed H_2_O molecule participated in O–O coupling to form *OOH (State 5) with a near-zero free uphill energy requirement. Please note that this step was different from the case of Fe-doped Ni oxyhydroxides (Ni_1−x_Fe_x_OOH), which are considered as the current most active non-noble electrocatalysts for OER under alkaline conditions and prefer the O–O coupling to occur at the low spin d^6^ Ni^4+^ rather than at high spin d^4^ Fe^4+^^54^. Whereas the low spin d^0^ W^6+^ stabilized the unpaired electron of the O and then assisted in the formation of O–O coupling. The above adjacent two steps did not then need to change their reactive sites and thereby the energy requirement was reduced. For the last step, the *OOH had to overcome a 1.67 -eV free energy barrier to achieve deprotonation into the O_2_ molecule (State 6), and consequently, the last step in the W-doped system is the Potential Determining Step (PDS) for OER. The overpotential was obtained based on the above results by subtracting the thermodynamic equilibrium potential (1.23 V for OER) from the highest free energy (1.67 eV) required for OER: ≅*η* = 1.67 eV/e −1.23 V = 0.44 V. Moreover, the spin states were also examined for the different states of the metal and intermediates as given in Table S6. Clearly, when compared with the high spin population (1.434) of Ni atom, a lower spin state of W atom was responsible for the stabilization of O–O bonding formation as discussed by Shin, Xiao, and Goddard^54^. As a contrast, in the case of pure Ni(OH)_2_, the first four states are all a result of endothermic reactions as shown in Fig. 5b, and step 4, the formation of *OOH_2_, is the PDS, which requires an uphill energy of 2.32 eV with a corresponding ƞ of 1.09 V. Furthermore, the O radical on Ni^4+^ was hard to form from State 3 to State 4, which induced an instability of O radical adsorption. The O–O–H formation (State 5) was an exothermic step and the final O–O coupling step needed an energy of 0.66 eV. The W-doped configurations, compared with bare Ni(OH)_2_, with a low spin population of 0.05 for the W atom showed it could stabilize the subsequent O–O coupling. As a result, the potential determining step of O radical formation on bare Ni(OH)_2_ is transferred to the deprotonation of *OOH. Also, the W element doped Ni(OH)_2_ sample gave the smallest overpotential at a relatively high current density of 80 mA/cm^2^ over all the Ni(OH)_2_-based OER catalysts in the literature. We also tried other dopant sites for W as shown in Supplementary Fig. 30. It was found that at the edge site, the dopant W is more energetically favorable than that of the interior W configuration. Note that although the PBE results cannot reproduce the exact experimental redox potentials due to the effect of the exchange-correlation functional in Ni oxyhydroxides systems, previous work showed that it has little influence on the knowledge of the concrete OER, especially at the PDS^55^. The K-point test of w-Ni(OH)_2_ systems for DFT calculations were carried out as shown in Supplementary Fig. 31. Meanwhile, we compared the dynamic processes of the PDS on w-Ni(OH)_2_ and Ni(OH)_2_ as shown in Supplementary Fig. 32. It was found that a lower kinetic energy barrier (2.05 eV) of the PDS on w-Ni(OH)_2_ was needed to be overcome when compared with that of PDS on pristine Ni(OH)_2_ (2.29 eV), meaning a faster electron transfer occurrence at this elementary step of OER. It agreed well with the steeper current response at low potential observed in the experiment.

## Discussion

In summary, single-atom W-doped Ni(OH)_2_ nanosheets were reported for the electrocatalytic water oxidation reaction. The w-Ni(OH)_2_ was synthesized from long-time ultrasonic exfoliation of w-Ni(OH)_2_-e, which was obtained via an alcohothermal method. Compared with the reference sample of pure layered Ni(OH)_2_, w-Ni(OH)_2_ gave an obvious enhanced OER performance: it only required an overpotential of 237 mV for reaching the current density of 10 mA/cm^2^. Moreover, at a high current density of 80 mA/cm^2^, the sample of w-Ni(OH)_2_ only needed a overpotential value of 267 mV with the corresponding Tafel slope measured to be 33 mV/dec. All the values were the best/highest level with respect to the current reported samples in the literatures. Based on DFT calculations, the w-Ni(OH)_2_ showed exothermic reactions for the first three steps of water oxidation: water adsorption, the formations of adsorbed OH^−^ group and the O radical. The site of d^0^ W^6+^ stabilized the O radical owing to the low spin population of 0.05. Most significantly, the following O–O coupling step was also generated at the W^6+^ site, and almost-zero eV of the Gibbs free energy was further required. This effect helped the W^6+^ ions boost the final water oxidation reaction of Ni(OH)_2_. This work can help us understand the effect of the HL on the water oxidation and provide a new direction to design doped OER catalysts.

## Methods

### Synthesis of pure Ni(OH)_2_-r

The Ni(OH)_2_ was prepared through an alcohothermal method. Typically, 0.4 g of NiCl_2_ was added into a 40 -mL ethanol solution. The mixture was stirred until a transparent solution was observed. The solution was then moved into a 50 -mL autoclave. The autoclave was then placed into an oven and treated in flowing air and heated at 150 °C for 12 h. Green powder of Ni(OH)_2_ was treated by 24 h ultrasound in an ethanol solution and then was collected by centrifuging the reaction mixture at a speed of 9000 r.p.m. for 10 min. The product was washed by water and ethanol four times and then was dried at 80 °C overnight. The obtained pure Ni(OH)_2_ was used as the reference sample and named as Ni(OH)_2_-r.

### Synthesis of w-Ni(OH)_2_-e

The w-Ni(OH)_2_-e was prepared through an alcohothermal method. At first, 1 g of WCl_6_ was added into 100 mL of ethanol to form a transparent solution. Then a certain amount of WCl_6_–ethanol solution was added into the NiCl_2_–ethanol solution (0.4g–40mL) by a pipette. After stirring for 15 min, the mixture was moved into a 50 -mL autoclave, which was then treated at 150 °C for 12 h in an oven. The product was collected by centrifuging the reaction mixture at a speed of 9000 r.p.m. for 10 min. After that, the powder was washed by water and ethanol four times and then dried at 80 °C overnight. The obtained W-doped Ni(OH)_2_ sample was named as w-Ni(OH)_2_-e.

### Synthesis of w-Ni(OH)_2_

The w-Ni(OH)_2_ was prepared through the exfoliation of the w-Ni(OH)_2_-e sample. Typically, 0.1 g of w-Ni(OH)_2_-e was added into 40 mL of ethanol and then the mixture was treated by ultrasound for 24 h. After that, the product was collected by centrifuging at a speed of 9000 r.p.m. for 10 min. The powder was washed by water and ethanol four times and then dried at 80 °C overnight. The obtained sample was named as w-Ni(OH)_2_.

### Structural characterization

Transmission electron microscopy (TEM) was performed on a FEI Tecnai G2 F20 electron microscope at an acceleration voltage of 200 kV. Spherical aberration-corrected electron microscopy was carried out on a JEM-ARM200F electron microscope. The field-emission scanning electron microscopy (FESEM) was observed under a SU8020 electron microscopy. X-ray diffraction (XRD) patterns of the samples were recorded on a Rigaku Smartlab-9kW instrument using Cu Kα X-ray (*λ* = 1.54186 Å) radiation at a scanning rate of 4 ^o^/min in the region of 2θ = 10–80^o^. X-ray photoelectron spectra (XPS) was acquired on a Kratos Axis Ultra DLD spectrometer with Al Kα (*hυ* = 1486.6 eV) as the excitation source. Fourier transform infrared reflectance (FTIR) and the Quasi FTIR spectra of samples were carried out on a Bruker V70 spectrometer. Diffuse reflectance ultraviolet–visible (UV–Vis) spectra of the studied samples were recorded in the air against BaSO_4_ in the region of 200–800 nm on a Perkin–Elmer Lambda 950 spectrophotometer. Raman analysis was carried out on a Renishaw InVia Raman spectrometer with a green line of an Ar-ion laser (514.53 nm) in micro-Raman configuration. XAFS experiments were performed at the 1W1B beamline of the Beijing Synchrotron Radiation Facility (BSRF). The storage ring runs at 2.0 GeV with a maximum electron current of about 450 mA. The energy range of the incident X-ray is tunable from 4 to 25 keV by fix-exit Si (111) double-crystal monochromator. The absorption edge of standard metal foils was used to calibrate the X-ray energy. Samples were ground into fine powers and then pressed into thin disks of 10 mm in diameter. W L3-edge XANES/EXAFS spectra were collected at room temperature in transmission mode. The data were processed using the IFEFFIT package^56^.

### OER electrode preparation

For the three samples studied and the reference RuO_2_, the preparation method was the same. Typically, 10 mg of the sample was added into a 50 μL of isopropanol and then 50 μL of nafion (Nafion 117 solution, Sigma-Aldrich) was dispersed into the solution. The mixture was first treated by the strenuous vibration of an oscillator. After that, the mixture was treated by ultrasound ca. 10 h to form the uniform ink, which was then dropwise applied onto glassy carbon electrodes with a mass loading of 0.2 mg cm^−2^. The prepared electrodes were naturally dried in air. For all the samples, four electrodes were prepared and the near results were used in our paper.

### Electrochemical characterizations

All of the electrochemical tests were performed at room temperature with the use of a typical three-electrode setup. The Pt (1 cm^2^) and Hg/HgO (1 M KOH) electrodes were used as the counter and reference electrodes, respectively. In all, 1 M KOH solution was used as the electrolyte. The electrocatalytic measurements were carried out on a Zennium Zahner electrochemical workstation. The measured potentials versus Hg/HgO were converted to the reversible hydrogen electrode (RHE) scale according to the following Nernst equation: E_RHE_ = E_Hg/HgO_ + 0.059 pH + E^o^_Hg/HgO_, where E_RHE_ is the converted potential versus RHE, E^o^_Hg/HgO_ = 0.098 at 25 °C, and E_Hg/HgO_ is the experimentally measured potential against the Hg/HgO reference. Before the test, the as-prepared anodes were activated by a chronopotentiometry scan with the 30 mA cm^−2^ current density for 2 h. The Tafel slopes were obtained from the polarization curves by plotting overpotential against log(current density). The steady-state activity was evaluated by chronopotentiometry measurements under different potentials. The ECSA was determined by measuring the capacitive current associated with double-layer charging from the scan rate CV-dependence. In our present work, the CV potential window was chosen to be 1.35 to 1.55 vs. RHE. The scan rates were 20, 40, 60, 80, and 100 mV s^–1^. The double-layer capacitance (C_dl_) was estimated Δ*j* = (*j*_charge_–*j*_off charge_) at 1.45 V vs. RHE against the scan rate. The linear slope is twice of the double-layer capacitance C_dl_. EIS measurements were carried out after the OER tests under the different potentials. The ZSim Demo software was used to fit the EIS results. The TOF was calculated using the below equation:^17,18^

7 $${\mathrm{TOF}} = \frac{{{\mathrm{J}} \ast {\mathrm{S}} \ast {\mathrm{\mu }}}}{{4 \ast {\mathrm{F}} \ast {\mathrm{n}}}}$$

where J (A cm^−2^) is the current density at a given overpotential (e.g., *η* = 250 mV), S is the surface area of the electrode (0.072 cm^2^), F is the Faraday constant (96,485 C mol^−1^), and *n* is the number of moles of reactive metal on the electrode. The μ is the Faradaic efficiency, which was determined from the total amount of charge Q (C) passed through the cell and the total amount of the produced O_2_, nO_2_ (mol): Faradaic efficiency = 4 F*nO_2_/Q, assuming the four electrons are needed to produce one oxygen molecule. Please note, we calculated the μ value to be ca. 100%. The metal content of Ni(OH)_2_ (55 wt.% Ni) and w-Ni(OH)_2_ (60 wt.% Ni and 5.5 wt.% W) was quantified by ICP–MS^33^. For the TOF calculation, the Ni and W are supposed to be the active sites for pure Ni(OH)_2_ and W-doped Ni(OH)_2_ samples, respectively.

### Computational details

All density functional theory calculations were realized by the Vienna ab initio simulation package (VASP)^57^. The exchange-correlation functional adopted the expression of Perdew–Burke–Ernzerhof (PBE) in the generalized gradient approximation (GGA) method^58^. Meanwhile, the Grimme method (DFT-D2)^59^ was introduced to consider the weak van der Waals’ interaction. The self-consistent calculations of single-electron wavefunction at ground state were terminated when the iterative convergence of energy and force fulfilled 10–5 eV and 0.01 eV/Å, respectively. Overall, 1 × 1 × 5 K-points mesh with a Gamma-centred Monkhorst-Pack scheme was sampled in the corresponding Brillouin zone on the reciprocal space (Fig. S28). A 15 Å lattice constant along the *z*-axis was set to avoid the layer image coupling caused by the periodic model. The detailed calculation results i.e., atomic Cartesian positions can be found in Table S5. Based on the standard hydrogen electrode model proposed by J. K Nørskov^60^, the Gibbs free energy change ΔG of oxygen evolution reaction (OER) on Ni(OH)_2_ and w-Ni(OH)_2_ were evaluated by the formula:

8 $$\Delta {\mathrm{G}} = \Delta {\mathrm{E}} + \Delta {\mathrm{ZPE}} - {\mathrm{T}}\Delta {\mathrm{S}}$$

where ΔE is the adsorption energy of OER intermediates and ΔZPE is their corresponding zero-point energy. The entropy contribution is simplified into its vibrational entropy of OER intermediates. Herein, although the PBE results cannot reproduce the exact experimental redox potentials due to the effect of the exchange-correlation functional in Ni oxyhydroxides systems, previous work showed that it has little influence on the knowledge of the concrete OER, especially at the PDS^55^. In order to determine the dynamic processes at the potential determining step, the climbing image nudged elastic band (CI-NEB)^61^ method was adopted to calculate the energy barriers of the transition state.

## Supplementary information

Supplementary Information

## Data Availability

The data that support the findings of this study are available on request from the corresponding authors.

## References

[1] Lewis NS, Nocera DG (2006). Powering the planet: chemical challenges in solar energy utilization. Proc. Natl. Acad. Sci. USA.

[2] Walter MG (2010). Solar water splitting cells. Chem. Rev..

[3] Reece SY (2011). Wireless solar water splitting using silicon-based semiconductors and earth-abundant catalysts. Science.

[4] Reier T, Nong HN, Teschner D, Schlögl R, Strasser P (2017). Electrocatalytic oxygen evolution reaction in acidic environments–reaction mechanisms and catalysts. Adv. Energy Mater..

[5] Suntivich J, May KJ, Gasteiger HA, Goodenough JB, Shao-Horn Y (2011). A perovskite oxide optimized for oxygen evolution catalysis from molecular orbital principles. Science.

[6] Mirzakulova E (2010). Electrode-assisted catalytic water oxidation by a flavin derivative. Nat. Chem..

[7] Anantharaj S (2018). Precision and correctness in the evaluation of electrocatalytic water splitting: revisiting activity parameters with a critical assessment. Energy Environ. Sci..

[8] He Q (2018). Highly defective Fe-based oxyhydroxides from electrochemical reconstruction for efficient oxygen evolution catalysis. ACS Energy Lett..

[9] Zhang JF (2018). Single-atom Au/NiFe layered double hydroxide electrocatalyst: probing the origin of activity for oxygen evolution reaction. J. Am. Chem. Soc..

[10] Liu KL (2018). The role of sctive oxide species for electrochemical water oxidation on the surface of 3d-metal phosphides. Adv. Energy Mater..

[11] Li T (2018). Atomic-scale insights into surface species of electrocatalysts in three dimensions. Nat. Catal..

[12] Fu HQ (2018). 1D/1D hierarchical nickel sulfide/phosphide nanostructures for electrocatalytic water oxidation. ACS Energy Lett..

[13] Gu C (2018). Synthesis of sub-2 nm iron-doped NiSe_2_ nanowires and their surface confined oxidation for oxygen evolution catalysis. Angew. Chem..

[14] Zhang B (2016). Homogeneously dispersed, multimetal oxygen-evolving catalysts. Science.

[15] Gao M (2014). Efficient water oxidation using nanostructured α-Nickel-Hydroxide as an electrocatalyst. J. Am. Chem. Soc..

[16] Duan L (2018). Direct synthesis and anion exchange of noncarbonate-intercalated NiFe-layered double hydroxides and the influence on electrocatalysis. Chem. Mater..

[17] Song F, Hu X (2014). Exfoliation of layered double hydroxides for enhanced oxygen evolution catalysis. Nat. Commun..

[18] Yan Z (2018). Anion insertion enhanced electrodeposition of robust metal hydroxide/oxide electrodes for oxygen evolution. Nat. Commun..

[19] Jiang J (2018). Atomic-level insight into super-efficient electrocatalytic oxygen evolution on iron and vanadium co-doped nickel (oxy)hydroxide. Nat. Commun..

[20] Li Z, Niu WH, Zhou L, Yang Y (2018). Phosphorus and aluminum codoped porous NiO nanosheets as highly efficient electrocatalysts for overall water splitting. ACS Energy Lett..

[21] Fang ZW (2017). Metallic transition metal selenide holey nanosheets for efficient oxygen evolution electrocatalysis. ACS Nano..

[22] Kakizaki H (2018). Evidence that crystal facet orientation dictates oxygen evolution intermediates on rutile manganese oxide. Adv. Funct. Mater..

[23] Huang JW (2018). A new member of electrocatalysts based on nickel metaphosphate nanocrystals for efficient water oxidation. Adv. Mater..

[24] Hung SF (2018). Unraveling geometrical site confinement in highly efficient iron-doped electrocatalysts toward oxygen evolution reaction. Adv. Energy Mater..

[25] Jin YS, Huang Sl, Yue X, Du HY, Shen PK (2018). Mo- and Fe-modified Ni(OH)_2_/NiOOH nanosheets as highly active and stable electrocatalysts for oxygen evolution reaction. ACS Catal..

[26] McAteer D (2018). Liquid exfoliated Co(OH)_2_ nanosheets as low-cost, yet high-performance, catalysts for the oxygen evolution reaction. Adv. Energy Mater..

[27] Corrigan DA (1987). The catalysis of the oxygen evolution reaction by iron impurities in thin-film nickel-oxide electrodes. J. Electrochem. Soc..

[28] Louie MW, Bell AT (2013). An investigation of thin-film Ni-Fe oxide catalysts for the electrochemical evolution of oxygen. J. Am. Chem. Soc..

[29] Smith RDL, Prevot MS, Fagan RD, Trudel S, Berlinguette CP (2013). Water oxidation catalysis: electrocatalytic response to metal stoichiometry in amorphous metal oxide films containing iron, cobalt, and nickel. J. Am. Chem. Soc..

[30] Trotochaud L, Young SL, Ranney JK, Boettcher SW (2014). Nickel–iron oxyhydroxide oxygen-evolution electrocatalysts: the role of intentional and incidental iron incorporation. J. Am. Chem. Soc..

[31] Friebel D (2015). Identification of highly active Fe sites in (Ni,Fe)OOH for electrocatalytic water splitting. J. Am. Chem. Soc..

[32] Chen JYC (2015). Operando analysis of NiFe and Fe oxyhydroxide electrocatalysts for water oxidation: detection of Fe^4+^ by mössbauer spectroscopy. J. Am. Chem. Soc..

[33] Görlin M (2016). Oxygen evolution reaction dynamics, faradaic charge efficiency, and the active metal redox states of Ni-Fe oxide water splitting electrocatalysts. J. Am. Chem. Soc..

[34] Stevens MB, Trang CDM, Enman LJ, Deng J, Boettcher SW (2017). Reactive Fe-sites in Ni/Fe (Oxy)hydroxide are responsible for exceptional oxygen electrocatalysis activity. J. Am. Chem. Soc..

[35] Görlin M (2017). Tracking catalyst redox states and reaction dynamics in Ni–Fe oxyhydroxide oxygen evolution reaction electrocatalysts: the role of catalyst support and electrolyte pH. J. Am. Chem. Soc..

[36] Diaz-Morales O, Ferrus-Suspedra D, Koper MTM (2016). The importance of nickel oxyhydroxide deprotonation on its activity towards electrochemical water oxidation. Chem. Sci..

[37] Bediako DK, Surendranath Y, Nocera DG (2013). Mechanistic studies of the oxygen evolution reaction mediated by a nickel-borate thin film electrocatalyst. J. Am. Chem. Soc..

[38] Subbaraman R (2011). Enhancing hydrogen evolution activity in water splitting by tailoring Li^+^-Ni(OH)_2_-Pt interfaces. Science.

[39] Danilovic N (2012). Enhancing the alkaline hydrogen evolution reaction activity through the bifunctionality of Ni(OH)_2_/metal catalysts. Angew. Chem. Int. Ed..

[40] Schouten KJP, van der Niet MJTC, Koper MTM (2010). Impedance spectroscopy of H and OH adsorption on stepped single-crystal platinum electrodes in alkaline and acidic media. Phys. Chem. Chem. Phys..

[41] Ledezma-Yanez I (2017). Interfacial water reorganization as a pH-dependent descriptor of the hydrogen evolution rate on platinum electrodes. Nat. Energy.

[42] Lin F, Boettcher SW (2014). Adaptive semiconductor/electrocatalyst junctions in water-splitting photoanodes. Nat. Mater..

[43] Zhang P (2018). Dendritic core-shell nickel-iron-copper metal/metal oxide electrode for efficient electrocatalytic water oxidation. Nat. Commun..

[44] Shin H, Xiao H, Goddard WA (2018). In silico discovery of new dopants for Fe-doped Ni oxyhydroxide (Ni_1–x_Fe_x_OOH) catalysts for oxygen evolution reaction. J. Am. Chem. Soc..

[45] Zhang J (2018). Cation vacancy stabilization of single-atomic-site Pt_1_/Ni(OH)_x_ catalyst for diboration of alkynes and alkenes. Nat. Commun..

[46] Zhang M (2017). Metal (Hydr)oxides@Polymer core-shell strategy to metal single atom materials. J. Am. Chm. Soc..

[47] Chen W (2018). Single tungsten atoms supported on MOF-derived N-doped carbon for robust electrochemical hydrogen evolution. Adv. Mater..

[48] Chen Y (2018). Single-atom catalysts: synthetic strategies and electrochemical applications. Joule.

[49] Hall DS, Lockwood DJ, Bock C, MacDougall BR (2014). Nickel hydroxides and related materials: a review of their structures, synthesis and properties. Proc. R. Soc. A.

[50] Aghazadeh M, Golikand AN, Ghaemi M (2011). Synthesis, characterization, and electrochemical properties of ultrafine β-Ni(OH)_2_ nanoparticles. Int. J. Hydrog. Energy.

[51] Hall DS, Lockwood DJ, Poirier S, Bock C, MacDougall BR (2012). Raman and infrared spectroscopy of α and β phases of thin nickel hydroxide films electrochemically formed on nickel. J. Phys. Chem. A.

[52] Yan JQ (2016). One-pot hydrothermal fabrication of layered β-Ni(OH)_2_/g-C_3_N_4_ nanohybrids for enhanced photocatalytic water splitting. Appl. Catal. B Environ..

[53] Burke MS (2015). Oxygen evolution reaction electrocatalysis on transition metal oxides and (oxy)hydroxides: activity trends and design principles. Chem. Mater..

[54] Shin H, Xiao H, Goddard WA (2018). In silico discovery of new dopants for Fe-doped Ni oxyhydroxide (Ni_1-x_Fe_x_OOH) catalysts for oxygen evolution reaction. J. Am. Chem. Soc..

[55] Xiao H, Shin H, Goddard WA (2018). Synergy between Fe and Ni in the optimal performance of (Ni,Fe)OOH catalysts for the oxygen evolution reaction. PNAS.

[56] Ravel B, Newville M (2005). ATHENA, ARTEMIS, HEPHAESTUS: data analysis for X-ray absorption spectroscopy using IFEFFIT. J. Synchrotron Rad..

[57] Kresse G, Furthmüller J (1996). Efficient iterative schemes for ab initio total-energy calculations using a plane-wave basis set. Phy. Rev. B.

[58] Perdew JP, Burke K, Ernzerhof M (1997). Generalized gradient approximation made simple. Phys. Rev. Lett..

[59] Grimme S (2006). Semiempirical GGA-type density functional constructed with a long-range dispersion correction. J. Comp. Chem..

[60] Nørskov JK, Rossmeisl J, Logadottir A, Lindqvist L (2004). Origin of the overpotential for oxygen reduction at a fuel-cell cathode. J. Phys. Chem. B.

[61] Henkelman G, Uberuaga BP, Jónsson H (2000). A climbing image nudged elastic band method for finding saddle points and minimum energy paths. J. Chem. Phys..

