# Supplementary material for: Single atom tungsten doped ultrathin α-Ni(OH)2 for enhanced electrocatalytic water oxidation
Source: Nat Commun. 2019 May 14;10:2149. doi: 10.1038/s41467-019-09845-z (PMC6517434; doi:10.1038/s41467-019-09845-z)
Supplement: Supplementary file 1 — Supplementary Information [file 41467_2019_9845_MOESM1_ESM.pdf]

Single atom tungsten doped ultrathin α-Ni(OH)$_2$ for enhanced electrocatalytic water oxidation

Yan et al.

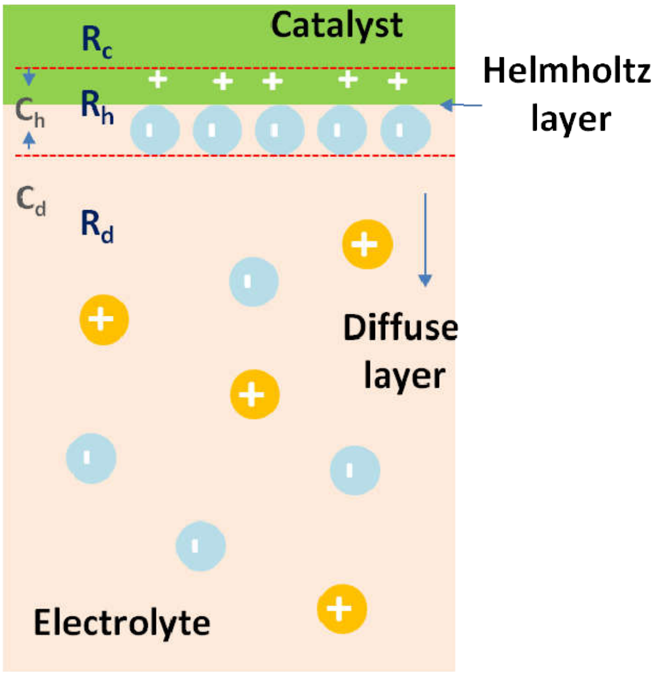

Supplementary Figure 1. Schematic diagram of the Helmholtz and diffuse layers in the electrocatalytic processes, the corresponding resistances i.e. catalyst (R_c), double electrode layer (R_h) and the electrolyte (R_d) are also shown.

Supplementary Table 1. The fitted results of the EIS plots in **Figure 1**b.

**Potential (V)**	**R_Ω (ohm/cm^2)**	**R_{ct} (ohm/cm^2)**	**C_d**
**1.2**	**10.8**	**277.4**	**$2.44*10^{-6}$**
**1.45**	**16.7**	**239.2**	**$3.08*10^{-6}$**
**1.6**	**17.5**	**168.6**	**$3.44*10^{-6}$**

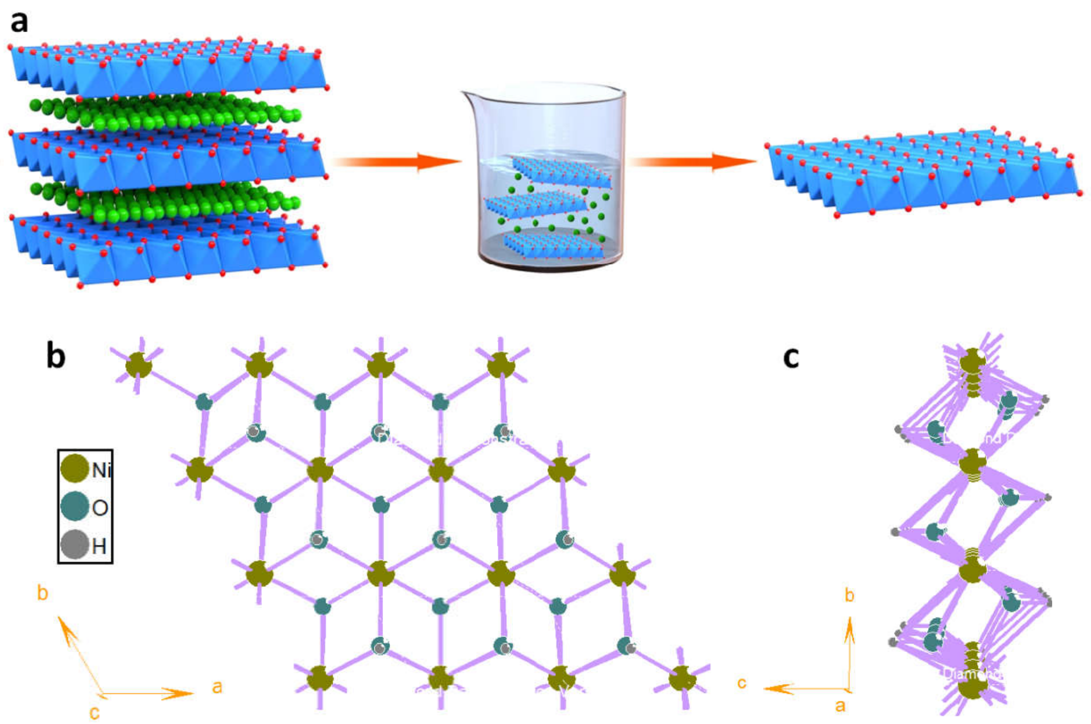

Supplementary Figure 2. (a) Schematic diagram of synthetic process of layered $Ni(OH)_2$; (b) and (c) the model of $Ni(OH)_2$ from z- and a-axis direction.

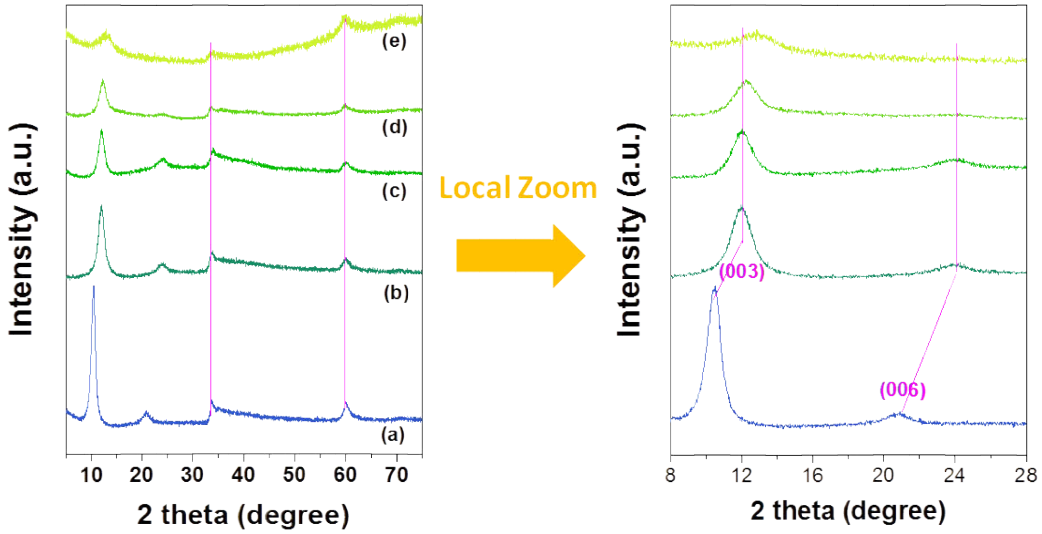

Supplementary Figure 3. XRD patterns of the w-Ni$(OH)_2$-e sample treated by ultrasound under the different time: (a) 6h, (b) 12h, (c) 18h and (d) 24h. The right gives the local zoom result at the 2 theta range from 8 to 28 degree.

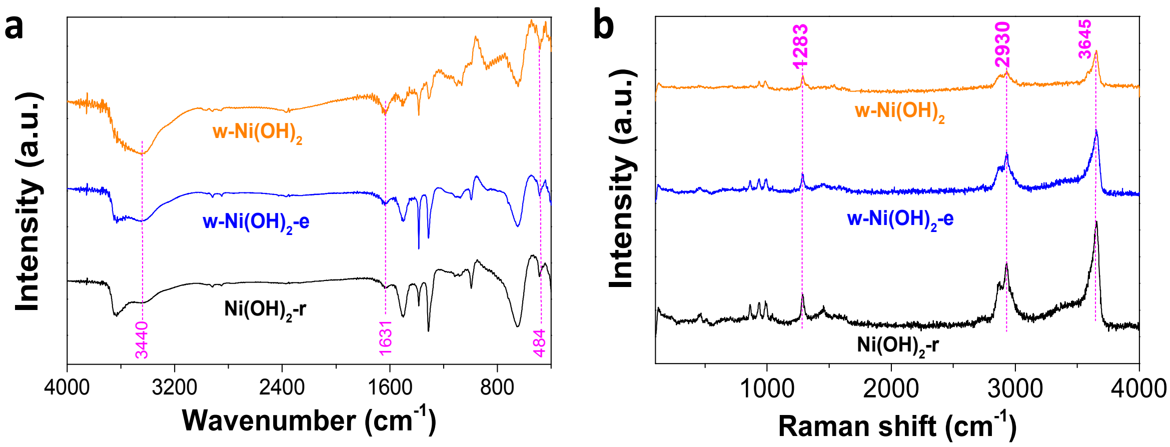

Supplementary Figure 4. (a) FTIR spectrum of the reference $Ni(OH)_2$-r, w-$Ni(OH)_2$-e and w-$Ni(OH)_2$ samples; (b) Raman spectra.

As is typical, the first peak at 484 cm^{-1} could be attributed to the stretching vibrational mode of the M-OH [50-51]; the 1631 cm^{-1} peak belonged to the free H_2O trapped by $Ni(OH)_2$ and the 3440 cm^{-1} peak was relative to the free H_2O. For the w-$Ni(OH)_2$ samples, the signal intensities of the three OH-based groups showed slightly stronger peak distinctions compared with the bare $Ni(OH)_2$ sample, suggesting that the exposed W sites were useful in aiding their adsorption.

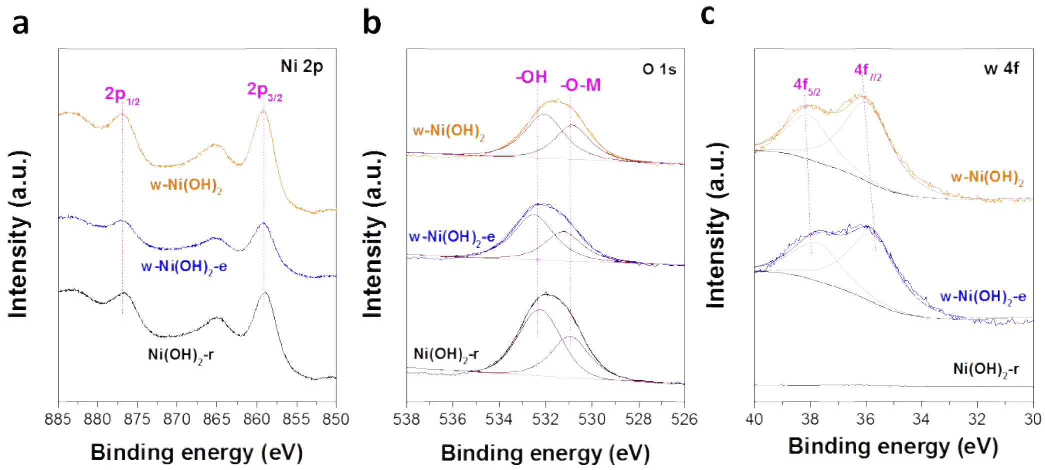

Supplementary Figure 5. XPS results of samples under study, (a) Ni 2p, (b) O 1s and (c) W 4f.

For Ni 2p (Figure S5a), the two main and typical signals i.e. 859 and 877 eV were detected. They were assigned to the Ni $2p_{3/2}$ and Ni $2p_{1/2}$ orbitals, respectively. In the case of O 1s in Figure S5b, the broad peak can be fitted to two signals, 530.9 and 532.4 eV, which belong to the O-M and O-H bonds, respectively. For the W doped samples, the Ni 2p and O 1s signals did not show any, suggesting that the W doping did not change the main electronic structure surrounding the Ni atom by any obvious means. Figure S5c shows the W 4f XPS signals. Two independent peaks of 35.7 and 37.8 eV belonged to the W $4f_{7/2}$ and W $4f_{5/2}$ of W^{6+}, respectively, confirming the high valance element doping. However, the w-Ni$(OH)_2$ sample showed a slightly higher binding energy shift compared to w-Ni$(OH)_2$-e. This may have originated from the slight change of the W doped Ni$(OH)_2$ structure after the exfoliation of the layered w-Ni$(OH)_2$-e.

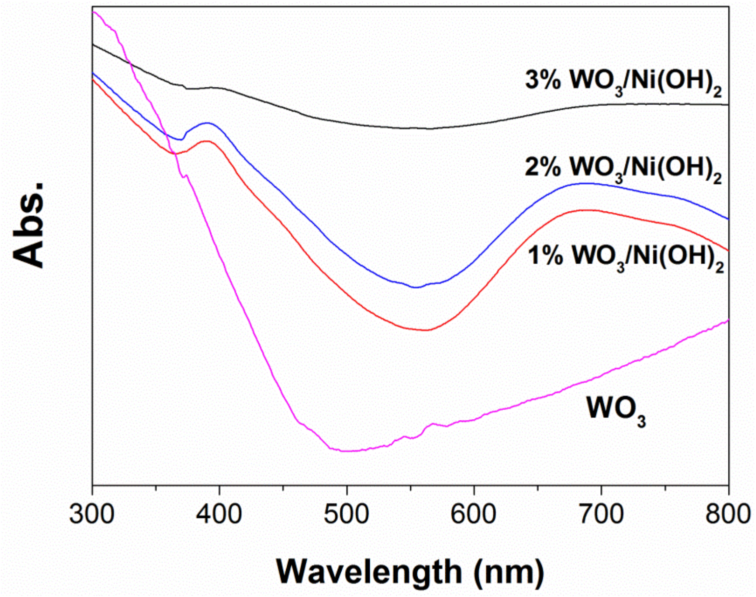

Supplementary Figure 6. UV-Vis absorption spectrum of the reference samples: WO_3, 1% 2% and 3% $WO_3/Ni(OH)_2$. Please note that, the red-shift of $WO_3/Ni(OH)_2$ samples compared with bare WO_3 can be assigned to the carrier migration across the interface between WO_3 and $Ni(OH)_2$ (Appl. Catal. B, 2014, 152, 280-288).

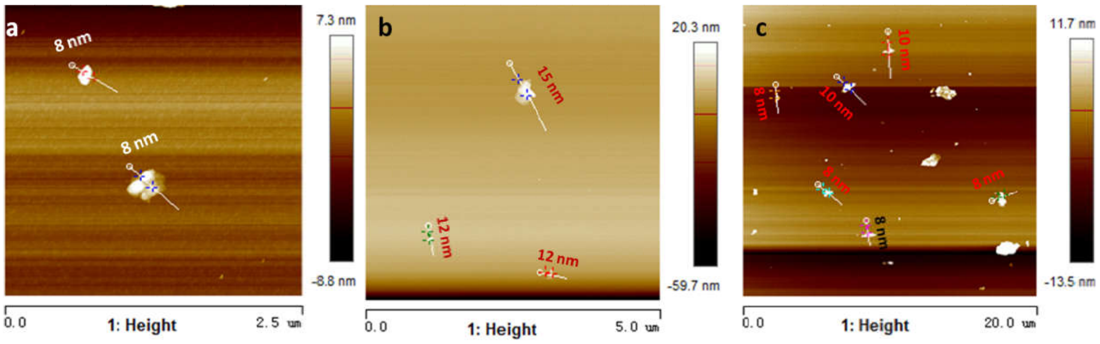

Supplementary Figure 7. AFM analysis of (a) $Ni(OH)_2$-r, (b) w-$Ni(OH)_2$-e and (c) w-$Ni(OH)_2$.

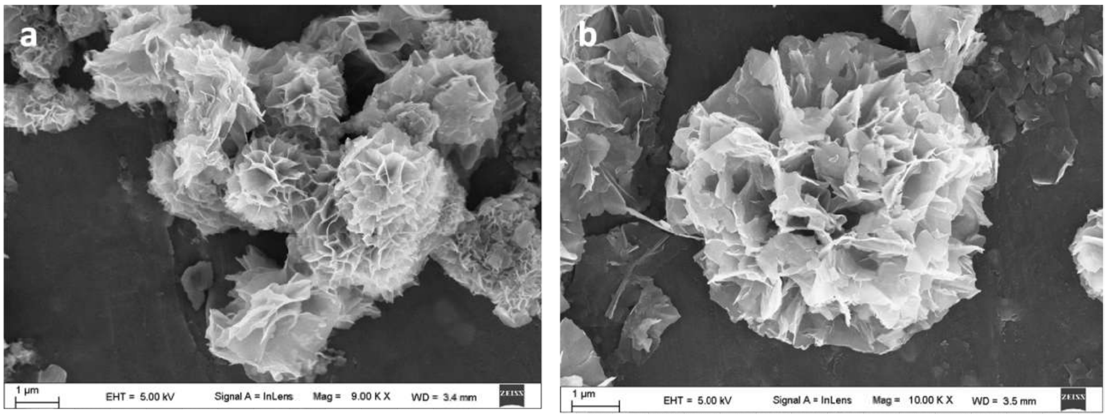

Supplementary Figure 8. SEM images of (a) the reference $Ni(OH)_2$-r sample and (b) w-$Ni(OH)_2$-e.

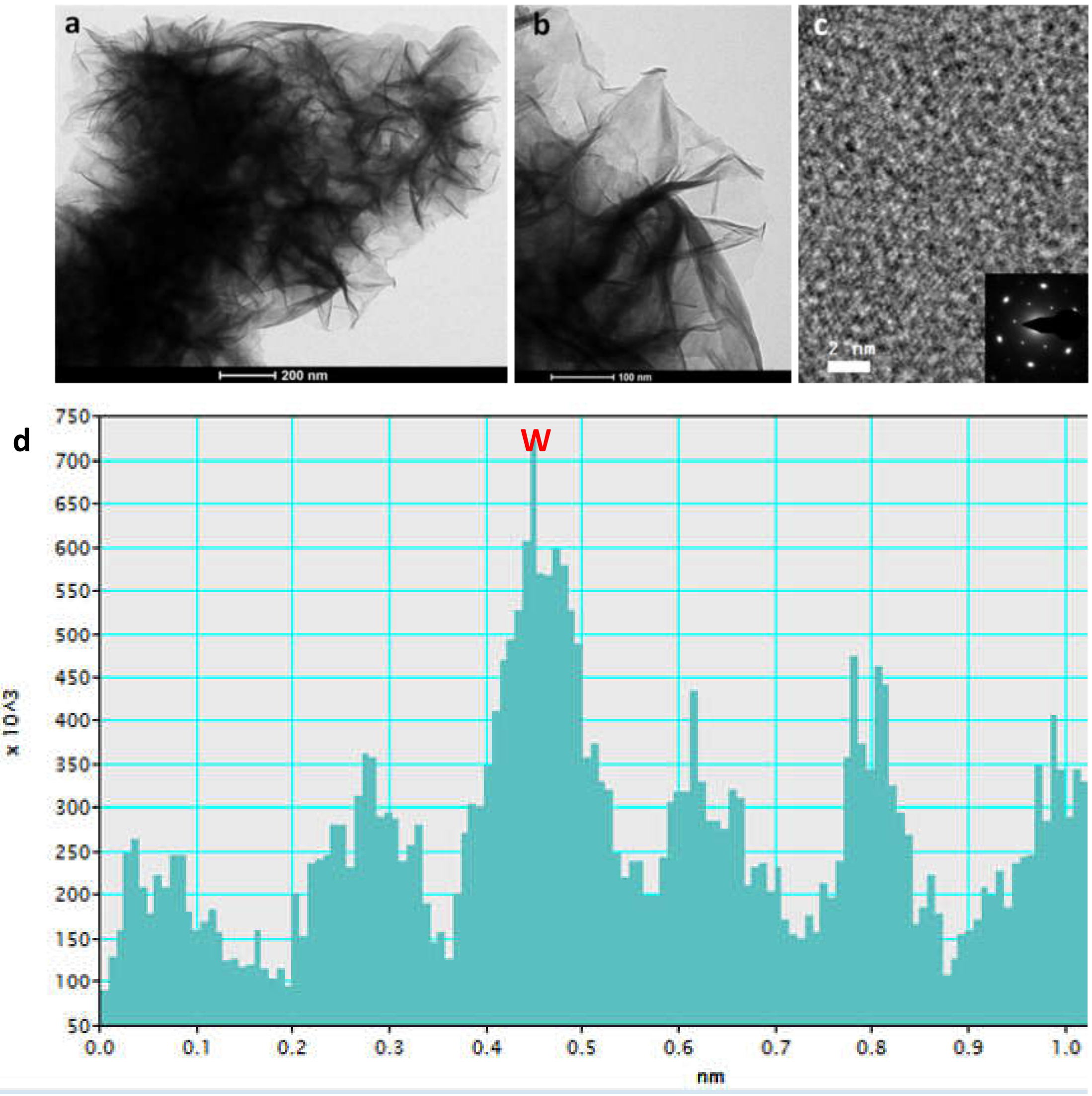

Supplementary Figure 9. TEM images of (a) the reference $Ni(OH)_2$-r sample, (b) w-$Ni(OH)_2$-e, (c) HRTEM image of w-$Ni(OH)_2$-e, the inset shows the corresponding EDS. (d) This result comes from Figure 3e.

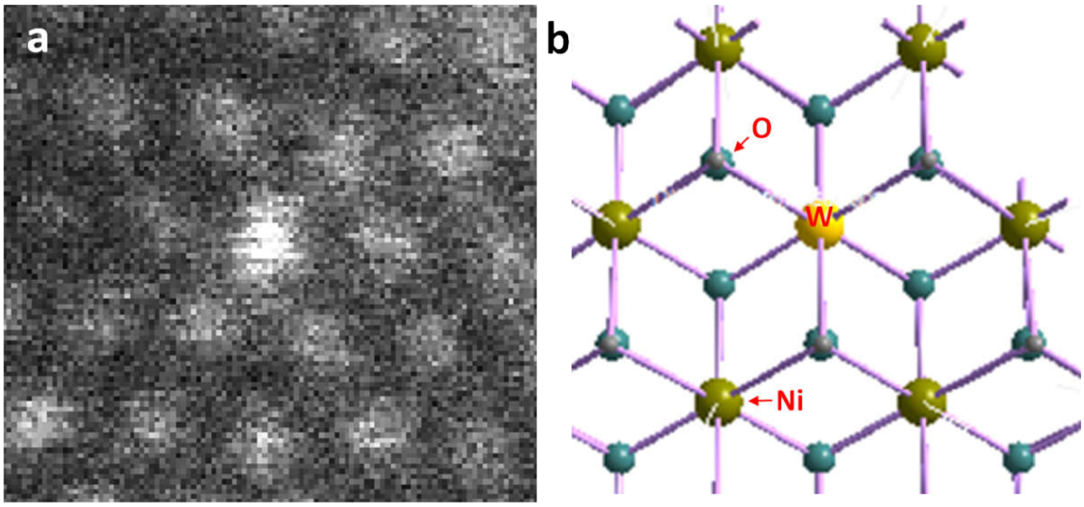

Supplementary Figure 10. (a) HADDF-STEM image of w-$Ni(OH)_2$ sample, (b) the mode of the W doped $Ni(OH)_2$.

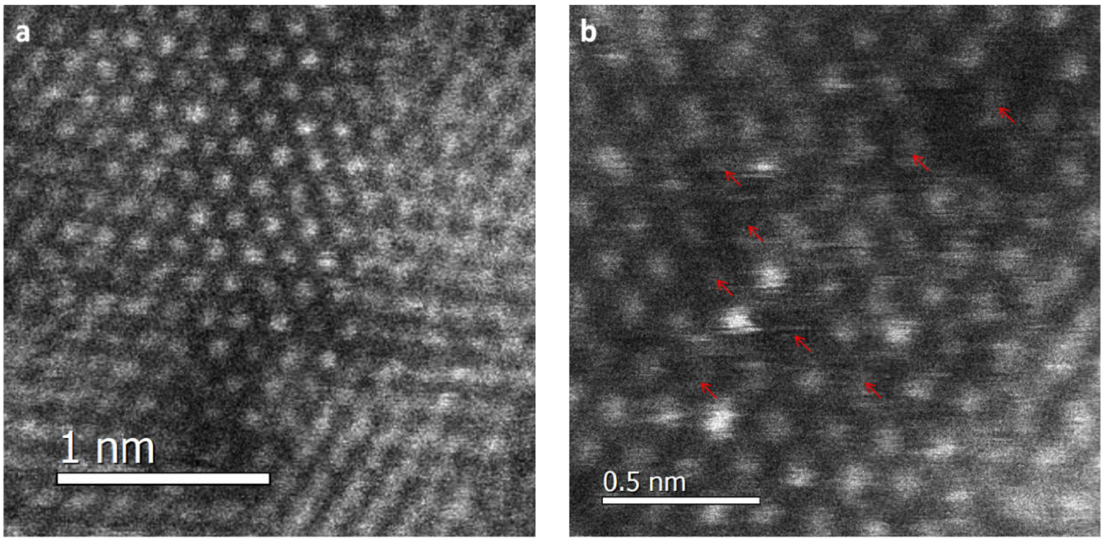

SupplementaryFigure 11. STEM images of pristine (a) and W doped (b) $Ni(OH)_2$ samples. The red arrows in b are corresponding disordered sites.

Supplementary Table 2. The atomic ratio of W to Ni of w-$Ni(OH)_2$ based samples.

Sample	ICP-MS	XPS	TEM-EDS
w-$Ni(OH)_2$-e	3.01 : 98	2.99 : 102	2.89 : 104
w-$Ni(OH)_2$	2.87 : 99.3	3.13 : 97.5	3.02 : 96.5

Supplementary Table 3. Comparison of catalytic performance of w-$Ni(OH)_2$ with reported Ni- based catalyst in 1.0 M KOH.

Materials	Support	Loading (mg cm^{-2})	$\eta_{@10\ mA\ cm-2}$ (mV)	$\eta_{@I\ mA\ cm-2}$ (mV)	Tafel slope (mv/dec)	$TOF_{@\eta/V}$ (s^{-1})	$ECSA_{@V}$ (mF cm^{-2})	Ref.
CS-NiFeCu	Ni foam	10.2	180	$250_{@248}$	33	-	54.24	S1
a-NiFe-OH/NiFeP	Ni foam	~1.8	199	$258_{@300}$	39	$0.036_{@0.25}$	$6.3_{@0.95}$	S2
Au/NiFe LDH	Ti mesh	2	237	$280_{@198}$	36	$0.11_{@0.28}$	$0.49_{@1.11}$	S3
NiFeV LDHs	Ni foam	2.8	192	$195_{@20}$	42	$0.04_{@0.25}$	$6.483_{@1.07}$	S4
$Ni_{0.75}V_{0.25}$-LDH	GC electrode	0.143	-	$350_{@27}$	50	-	$0.27_{@1.25}$	S5
NiCo LDH	Carbon paper	~0.17	367	$300_{@683}$	40	-	-	S6
NiFeCr LDH	Carbon paper	0.2	-	$225_{@25}$	69	-	$1.176_{@0.25}$	S7
NiFe LDH@NiCoP/NF	Ni foam	2	220	-	48.6	-	18.07	S8
NiFe LDH/r-GO	Ni foam	0.25	206	-	39	$0.987_{@0.3}$	-	S9
NiFe LDH/r-GO	GC electrode	0.25	230		42	-	-	S10
Holey $Ni(OH)_2$	GC electrode	-	335	-	65	$1.52 \times 10^{-2}_{@0.35}$	-	S11
NiFeRu-LDH	Ni foam	-	225	-	-	-	-	S12
NiFe−OOH	Ni foam	-	-	$270_{@240}$	40	$0.146_{@0.4}$	-	S13
$Ni_3Fe_{0.5}V_{0.5}$-OH	carbon fiber paper	-	200	$264_{@100}$	39	$0.574_{@0.3}$	-	S14
**w-$Ni(OH)_2$**	**GC electrode**	**0.2**	**237**	**$267_{@80}$**	**33**	**$0.74_{@0.25}$**	**$5_{@1.45}$**	**This work**

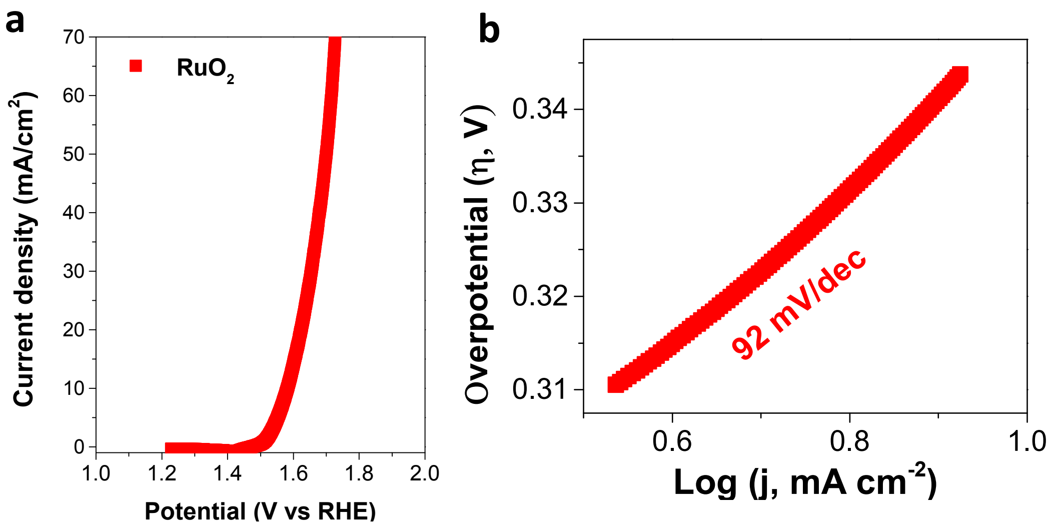

Supplementary Figure 12. (a) Polarization curve of RuO_2 and (b) the corresponding Tafel slope.

Supplementary Table 4. The fitted results of the EIS plots in **Figure 4**d.

**Sample**	**R_{Ω} (ohm)**	**R_{ct} (ohm)**
$Ni(OH)_2$-r	5.72	446.7
w-$Ni(OH)_2$-e	5.46	291.5
w-$Ni(OH)_2$	5.29	160

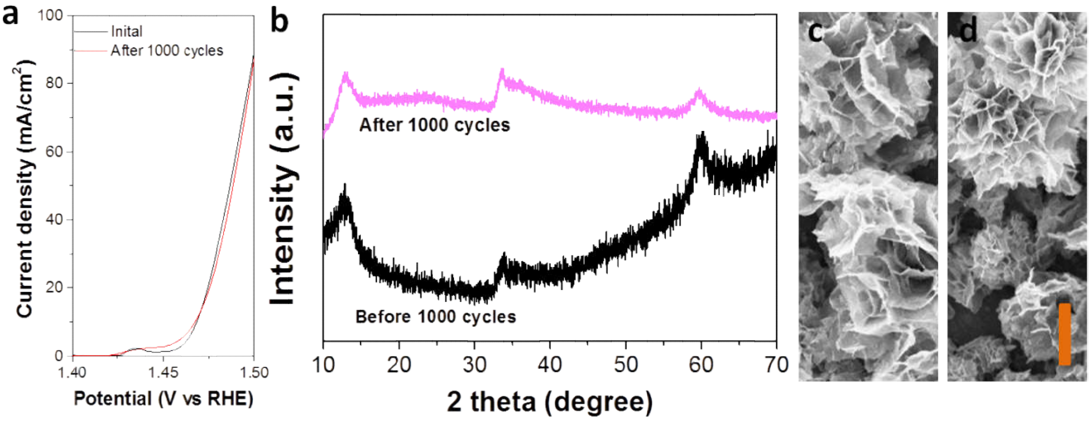

Supplementary Figure 13. (a) The polarization curves of w-$Ni(OH)_2$ sample after 1000 cycles. (b) XRD patterns of the w-$Ni(OH)_2$ sample before and after the 1000 cycles. (c) and (d) SEM images of the w-$Ni(OH)_2$ before and after 1000 cycles, respectively. Scale bar, 100 nm

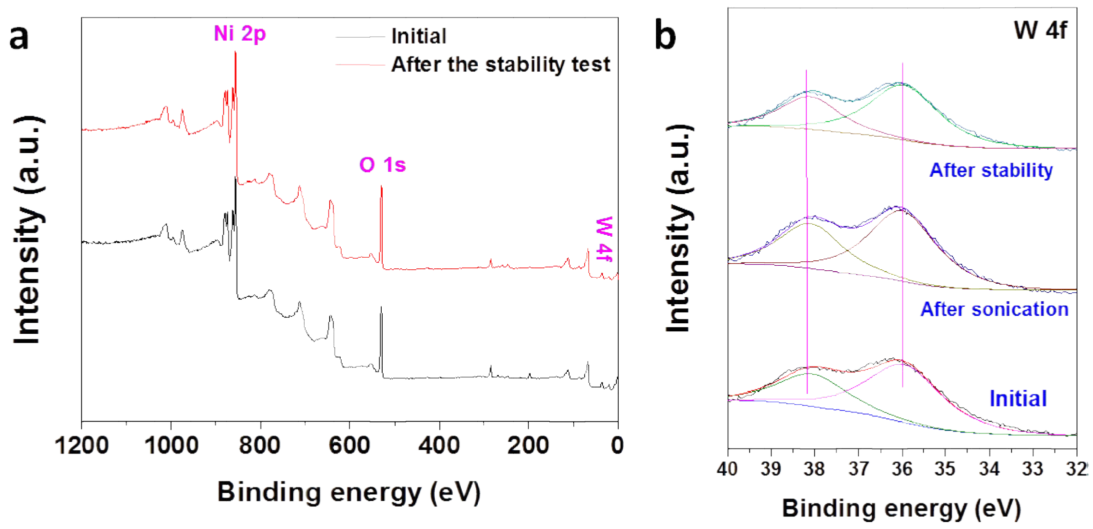

SupplementaryFigure 14. XPS results: (a) the wide spectrum of initial and after the stability tests; (b) W 4f spectra of the initial, after 10 h sonication and after long-time stability test .

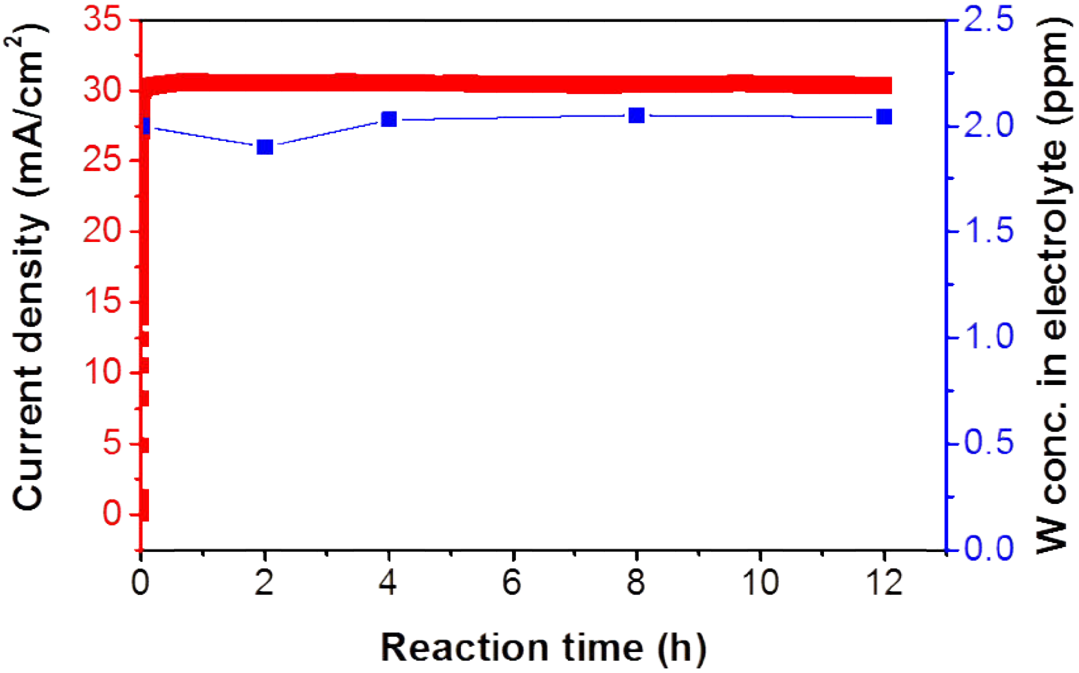

Supplementary Figure 15. Galvanostatic polarization curve and ICP-MS of w-$Ni(OH)_2$ in 1M KOH vs. time,

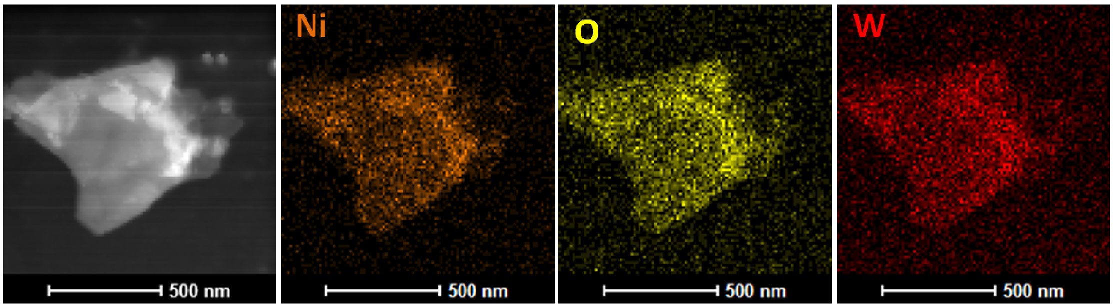

Supplementary Figure 16. The element mapping results after the long-time stability test.

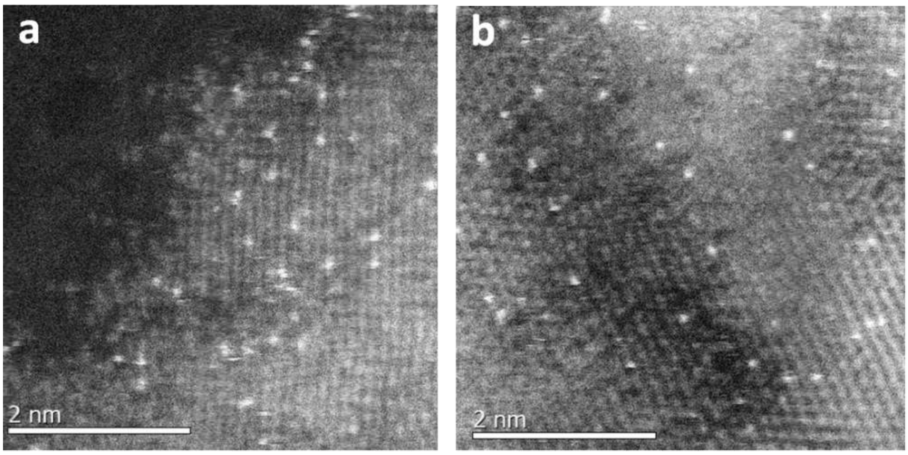

Supplementary Figure 17. The STEM results of w-$Ni(OH)_2$ sample (a) before and (b) after the long-time stability test.

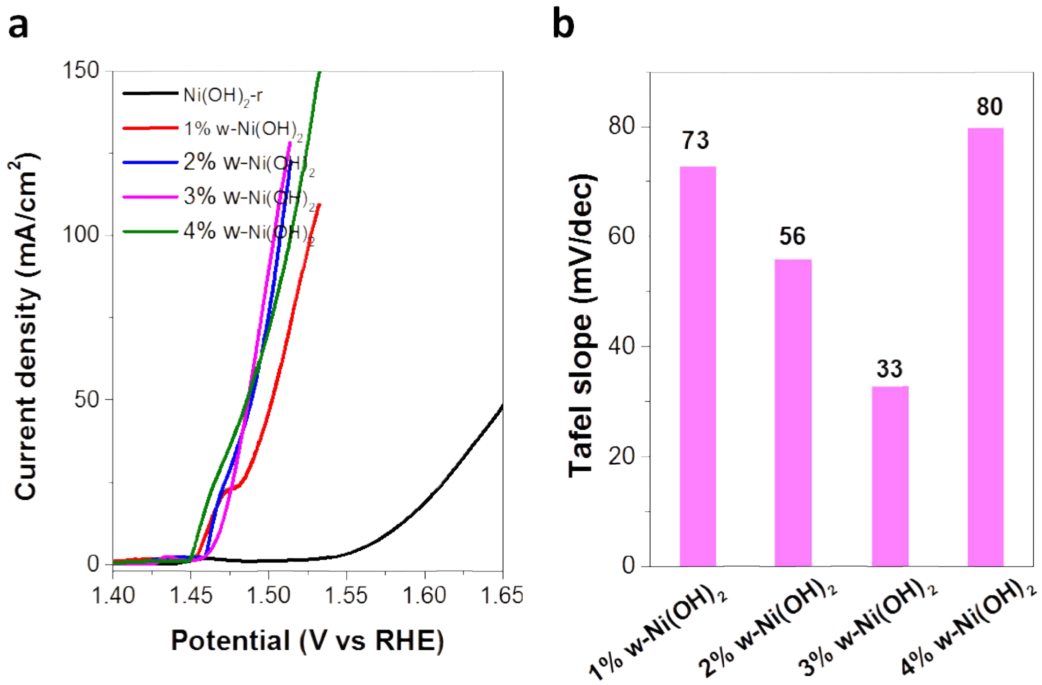

Supplementary Figure 18. (a) The polarization curves of w-$Ni(OH)_2$ samples with the different W element doping. (b) The corresponding Tafel slopes of the studied samples.

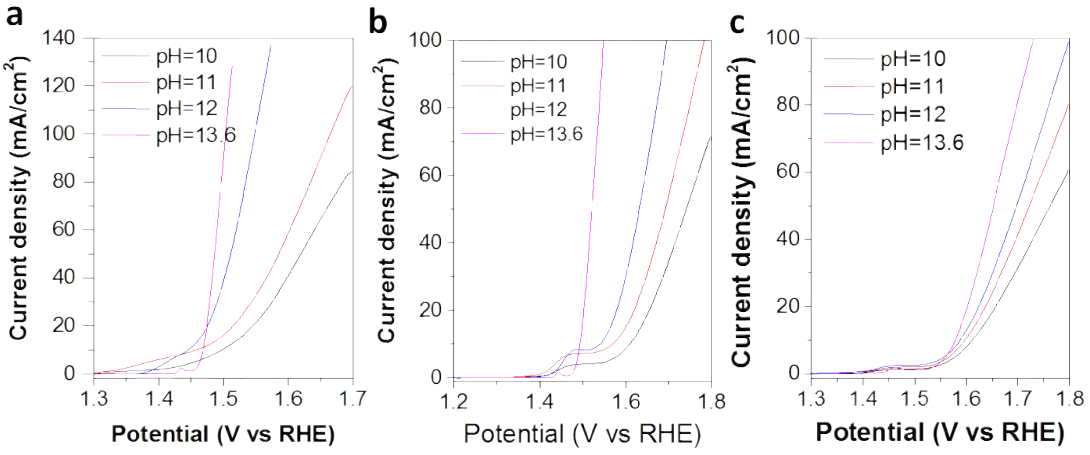

Supplementary Figure 19. The polarization curves of (a) $Ni(OH)_2$-r, (b) w-$Ni(OH)_2$-e and (c) w-$Ni(OH)_2$ sample under the different pH solution. The KOH solution was used for changing the pH.

More free OH^- ions are thought to have aided adsorption onto the reaction sites and assisted in the carrier interfacial migration, promoting the oxygen evolution. Moreover, the w-$Ni(OH)_2$ exhibits the best performance under the four pH conditions, further suggesting its superiority for water oxidation in contrast to the reference catalysts.

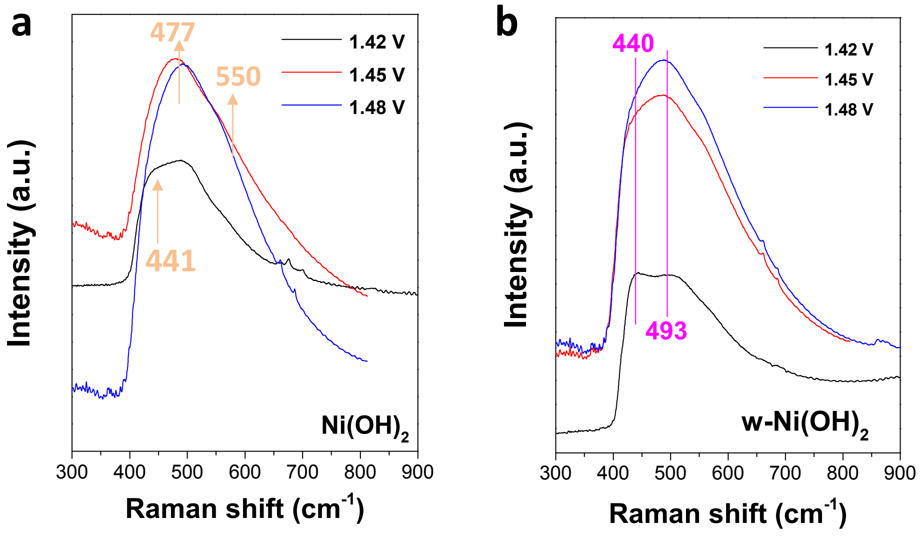

SupplementaryFigure 20. *In situ* Raman spectra collected on (a) $Ni(OH)_2$ and (b) w-$Ni(OH)_2$ electrodes under three potentials (1.42, 1.45 and 1.48 V vs. RHE) in 1 M KOH.

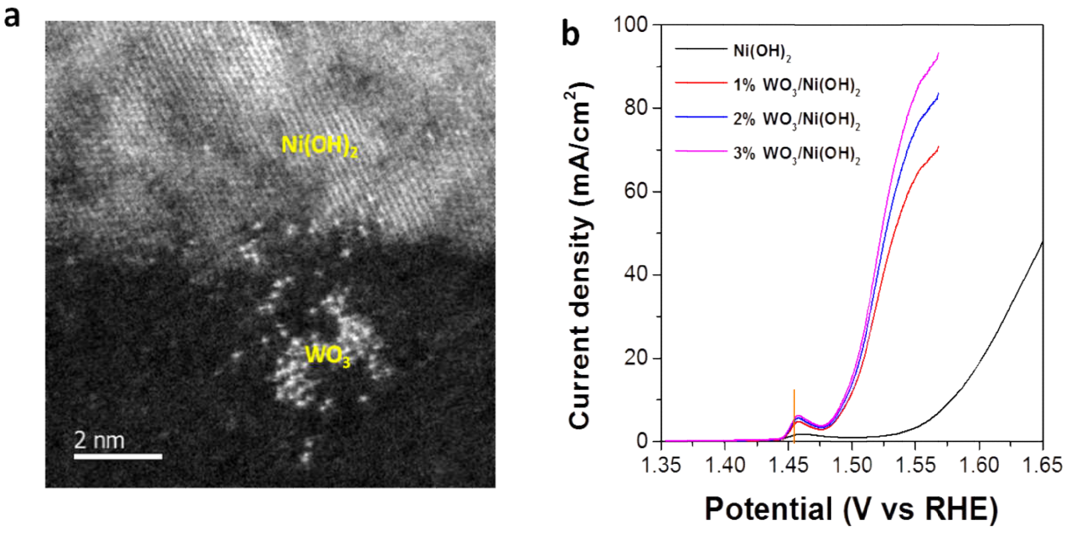

Supplementary Figure 21. (a) STEM image of 3% $WO_3/Ni(OH)_2$ sample; (b) the OER performance of $WO_3/Ni(OH)_2$ and $Ni(OH)_2$.

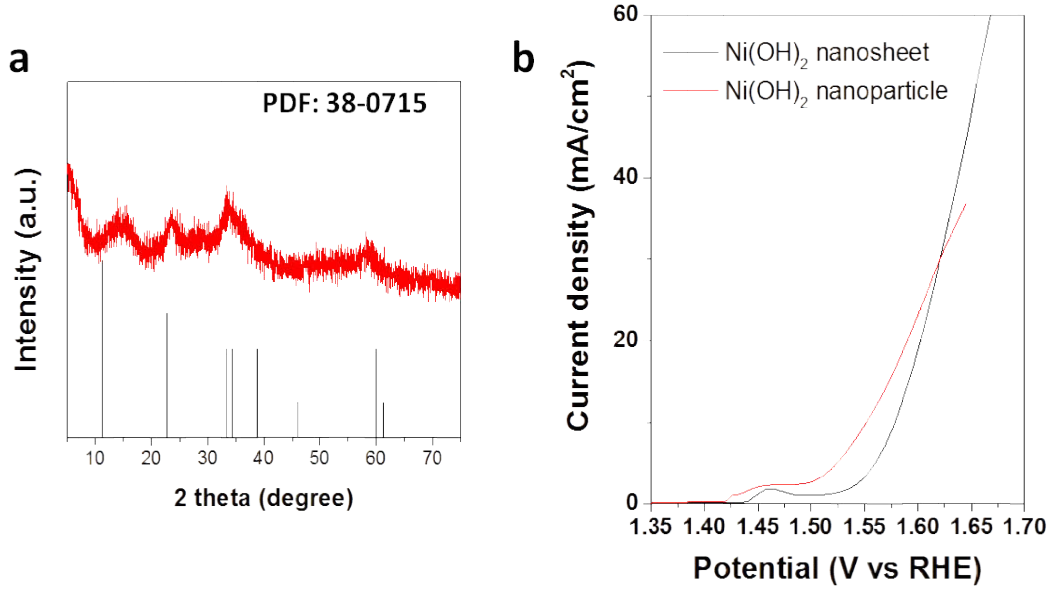

Supplementary Figure 22. (a) XRD of 3% $WO_3/Ni(OH)_2$ sample; (b) the OER performance of $WO_3/Ni(OH)_2$ and $Ni(OH)_2$. Please note that the $Ni(OH)_2$ nanoparticles shows the low XRD intensity, suggesting its low crystallinity. Many defect sites on its surface may be responsible for the similar OER performance with our $Ni(OH)_2$ nanosheet.

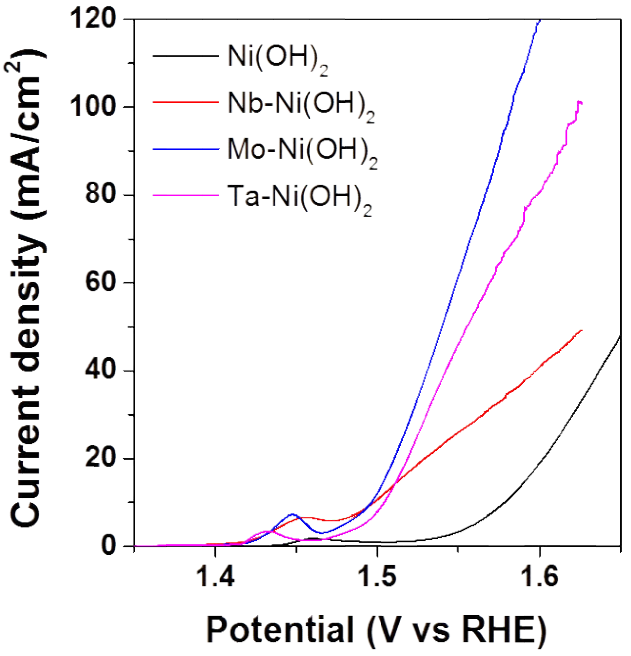

Supplementary Figure 23. OER performance of Nb, Mo and Ta doped $Ni(OH)_2$. 1M KOH is the electrolyte.

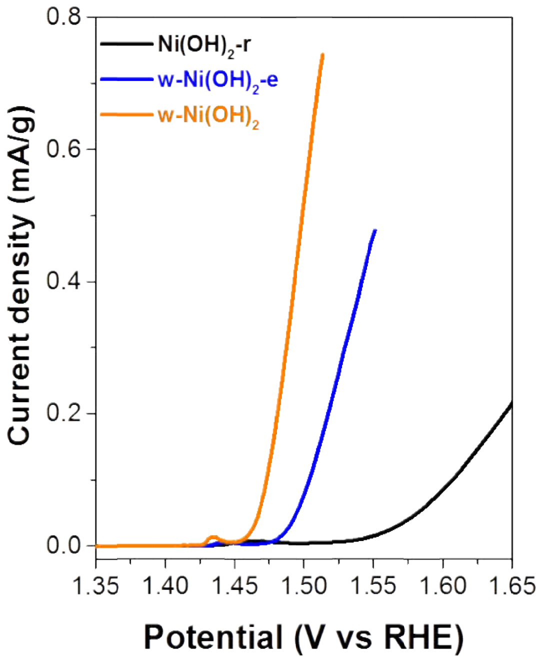

Supplementary Figure 24. The specific activity of the samples under study.

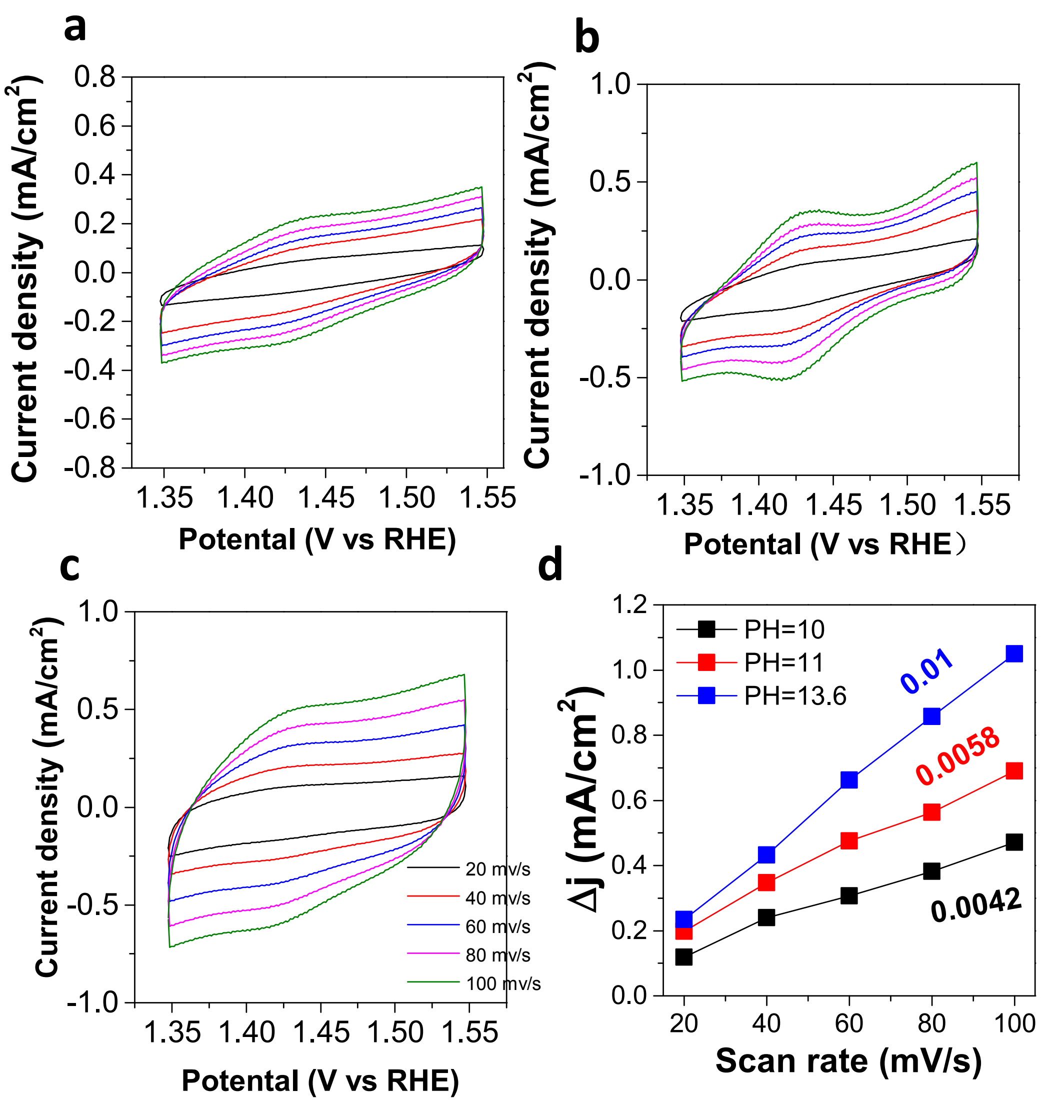

Supplementary Figure 25. Typical cyclic voltammetry curves of w-$Ni(OH)_2$ electrode with different scan rates in three pH solution conditions: (a) pH=10, (b) pH=11 and (c) pH=13.6. (d) Δj at potential of 1.45 V (vs RHE) of w-$Ni(OH)_2$ plotted against scan rates.

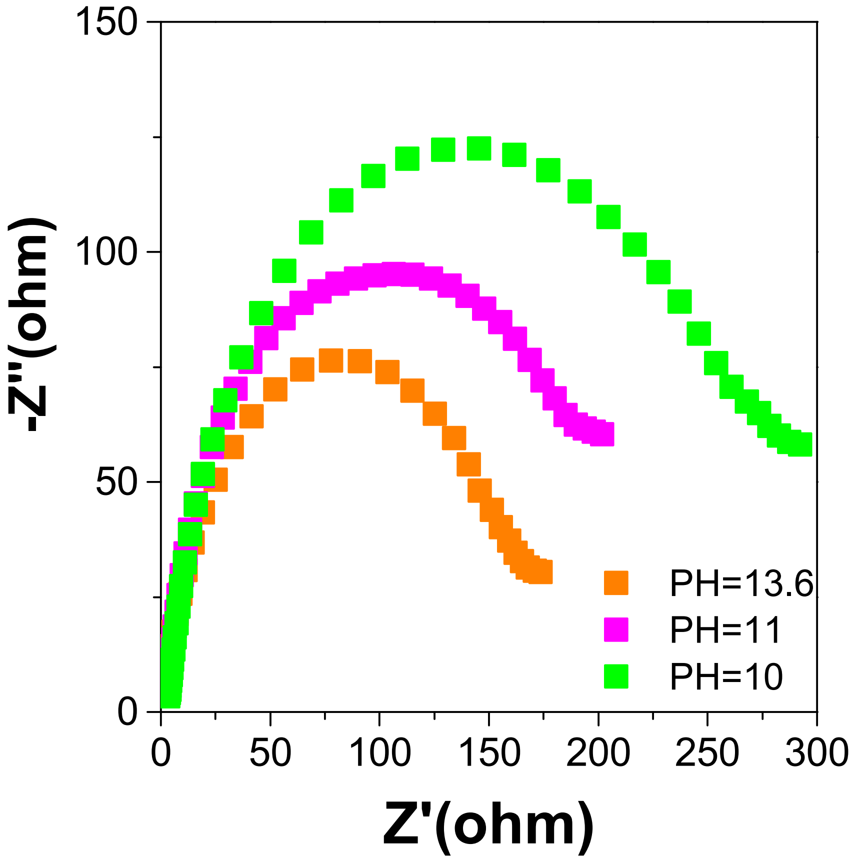

Supplementary Figure 26. EIS plots of w-$Ni(OH)_2$ electrode under the chosen three pH solution conditions, 13.6, 11 and 10.

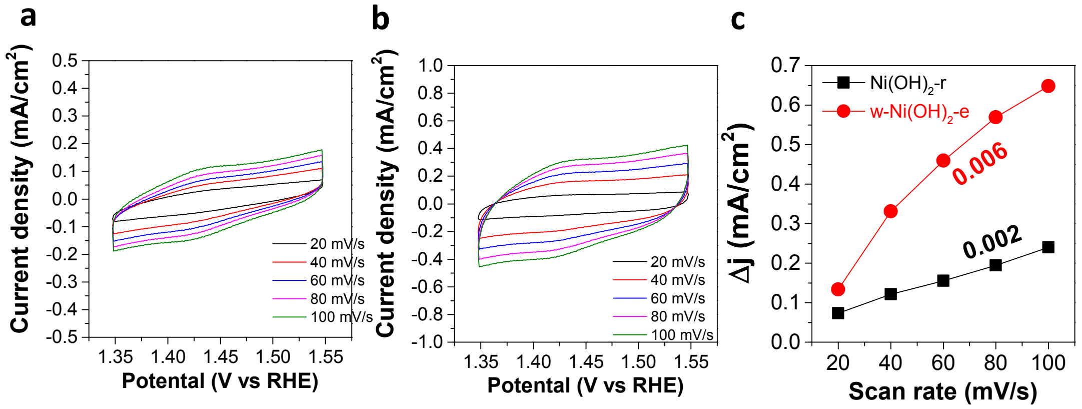

Supplementary Figure 27. Cyclic voltammetry curves of (a) $Ni(OH)_2$-r and (b) w-$Ni(OH)_2$-e electrodes with different scan rates in pH=13.6 solution conditions, (c) the corresponding Δj at potential of 0.45 V (vs Ag/AgCl) of $Ni(OH)_2$-r and w-$Ni(OH)_2$-e electrodes plotted against scan rates.

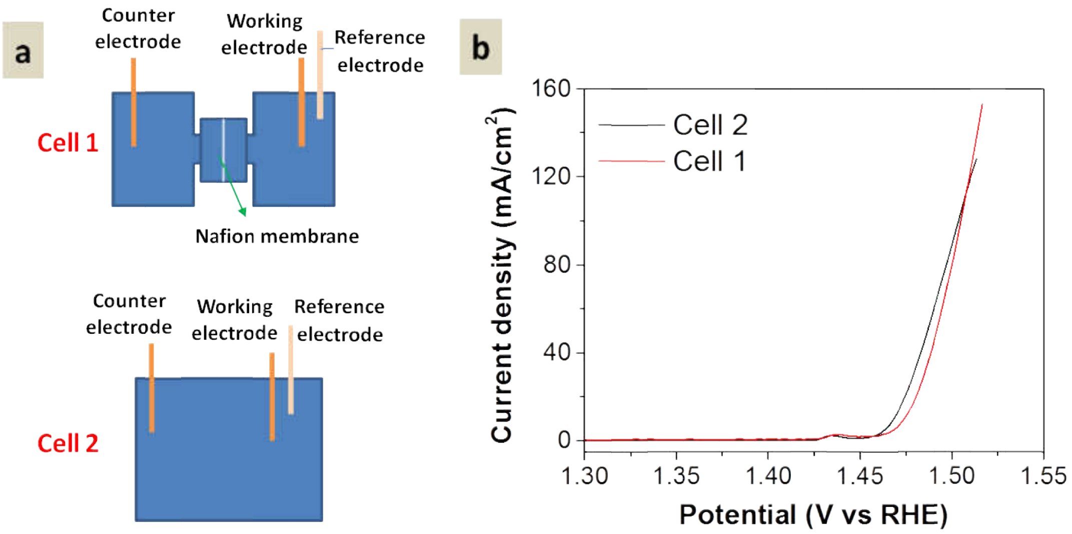

Supplementary Figure 28. (a) The mode of the electrocatalytic cells for the water splitting, cell 1 is similar to the real electrolyzer and the cell 2 is the experimental electrolyzer. (b) The comparison of the OER performance from the two different cells.

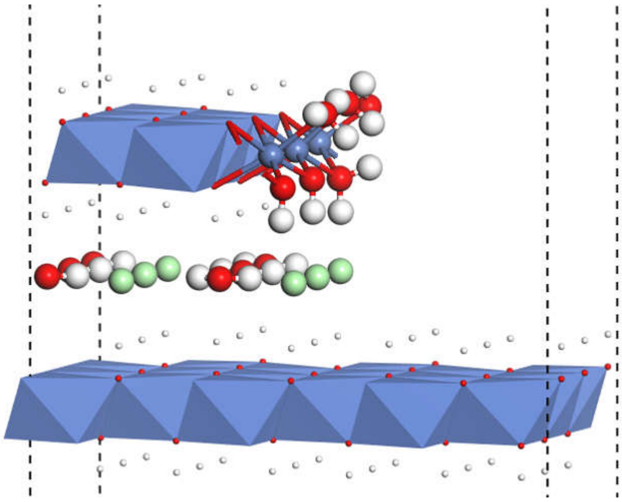

Supplementary Figure 29. Model of alpha-$Ni(OH)_2$ catalyst for DFT calculations. Two layers intercalated with Cl^- and H_2O are shown.

Supplementary Table 6. The spin population of the edge Ni and W atoms at different states (1~6).

mag(μ_B)	Ni	W
State 1	1.443	0.012
State 2	1.450	0.033
State 3	1.438	0.036
State 4	1.437	-0.004
State 5	1.434	0.005
State 6	1.436	0.005

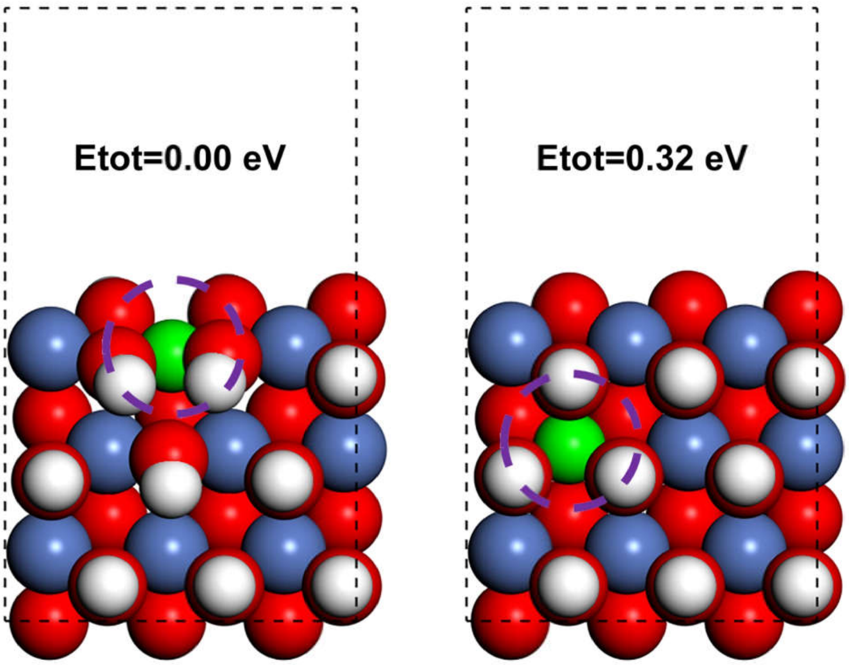

Supplementary Figure 30. Dopant site search of W dopant in $Ni(OH)_2$ surface. Herein, only the first and second metal layer were taken into consideration due to that the third metal layer was constrained to simulate its bulk phase.

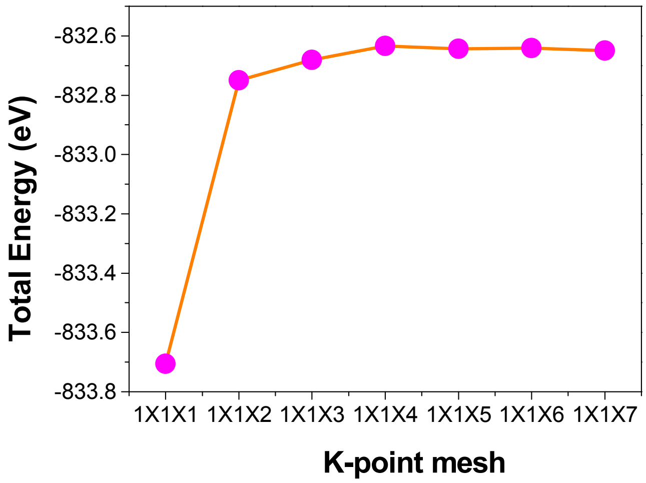

Supplementary Figure 31. K-point test of w-$Ni(OH)_2$ systems in DFT calculations.

We conducted the K-point test as given in Figure S28 and found that 1×1×5 K-point mesh was better to converge, thus we chose the 1×1×5 K-point mesh for all the DFT studies.

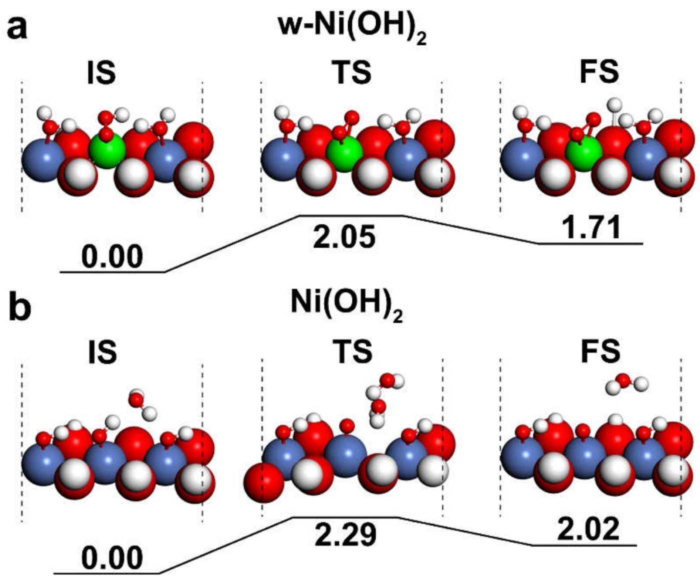

Supplementary Figure 32. The dynamic energy barriers of the transition state for w-$Ni(OH)_2$ and $Ni(OH)_2$ at the potential determining step.

Supplementary Table 7. The optimized atomic Cartesian positions of pristine $Ni(OH)_2$ and w-doped $Ni(OH)_2$.

Pristine $Ni(OH)_2$		
1.0		
16.3799991608	0.0000000000	0.0000000000
0.0000000000	30.0000000000	0.0000000000
0.0000000000	0.0000000000	9.3599996567
Cl H Ni O		
6 72 27 66		
Cartesian		
2.730054372	16.992899179	8.687296210
8.189999580	16.992899179	8.687296210
2.730054372	16.992899179	5.567234026
8.189999580	16.992899179	5.567234026
2.730054372	16.992899179	2.447265569
8.189999580	16.992899179	2.447265569
4.541682191	11.127299666	8.959579039
0.918262773	22.858500481	8.959579039
5.439470229	15.077700019	7.394118748
0.020474998	18.908100128	7.394118748
1.746599269	11.102999747	7.397394734
3.429009383	22.846493125	7.411963720
2.712036556	15.005400181	8.942637306
2.748072432	18.980399966	8.942637306
0.793283351	16.989900470	0.230630387
10.001791423	11.127299666	8.959579039
6.252577779	22.871729136	8.947071491
10.899578973	15.077700019	7.394118748
5.394218587	18.868957758	7.385272137
7.206544478	11.102999747	7.397394734
8.171981520	15.005400181	8.942637306
7.858602009	18.946396708	8.966594625
4.666825453	16.995899677	0.230630387
6.253228499	16.989900470	0.230630387
15.461736143	11.127299666	8.959579039
16.359523693	15.077700019	7.394118748
12.666489199	11.102999747	7.397394734
13.631926241	15.005400181	8.942637306
4.541682191	11.127299666	5.839516855
0.918262773	22.858500481	5.839516855
5.439470229	15.077700019	4.274150291

0.020474998	18.908100128	4.274150291
1.746599269	11.102999747	4.277426277
3.641021808	22.855551839	4.274590195
2.712036556	15.005400181	5.822575122
2.748072432	18.980399966	5.822575122
0.793283351	16.989900470	6.470661255
10.001791423	11.127299666	5.839516855
6.281245582	22.845422029	5.891052817
10.899578973	15.077700019	4.274150291
5.460124364	18.849502802	4.281836193
7.206544478	11.102999747	4.277426277
8.171981520	15.005400181	5.822575122
8.145609544	19.035932422	5.954585291
4.666825453	16.995899677	6.470661255
6.253228499	16.989900470	6.470661255
15.461736143	11.127299666	5.839516855
16.359523693	15.077700019	4.274150291
12.666489199	11.102999747	4.277426277
13.631926241	15.005400181	5.822575122
4.541682191	11.127299666	2.719547840
0.918262773	22.858500481	2.719547840
5.439470229	15.077700019	1.154181555
0.020474998	18.908100128	1.154181555
1.746599269	11.102999747	1.157363953
3.502551235	22.827438712	1.171034024
2.712036556	15.005400181	2.702606386
2.748072432	18.980399966	2.702606386
0.793283351	16.989900470	3.350599071
10.001791423	11.127299666	2.719547840
6.438652447	22.765132785	2.668953612
10.899578973	15.077700019	1.154181555
5.503994508	18.869529963	1.116896646
7.206544478	11.102999747	1.157363953
8.171981520	15.005400181	2.702606386
8.266183121	18.833511472	2.720647180
4.666825453	16.995899677	3.350599071
6.253228499	16.989900470	3.350599071
15.461736143	11.127299666	2.719547840
16.359523693	15.077700019	1.154181555
12.666489199	11.102999747	1.157363953
13.631926241	15.005400181	2.702606386
8.708570184	22.712236047	1.632053401
9.114302138	20.198165774	2.458807393

8.842317790	22.421908379	5.606770686
9.689078741	21.701937318	4.553379247
8.899834015	22.215600014	8.767298434
9.577642092	21.435747743	7.586156667
0.880424970	13.120199740	8.973151059
4.516407603	20.856601596	8.963874307
3.613591496	13.102200329	7.414804544
1.846353469	20.883600712	7.414804544
6.340370300	13.120199740	8.973151059
9.073536705	13.102200329	7.414804544
7.176045092	20.849812031	7.441701993
11.800479044	13.120199740	8.973151059
14.533645448	13.102200329	7.414804544
0.880424970	13.120199740	5.853182044
4.541044164	20.844338536	5.838616405
3.613591496	13.102200329	4.294835808
1.846353469	20.883600712	4.294835808
6.340370300	13.120199740	5.853182044
9.073536705	13.102200329	4.294835808
7.248870566	20.835347772	4.371216707
11.800479044	13.120199740	5.853182044
14.533645448	13.102200329	4.294835808
0.880424970	13.120199740	2.733213587
4.561863297	20.826750398	2.742136352
3.613591496	13.102200329	1.174773624
1.846353469	20.883600712	1.174773624
6.340370300	13.120199740	2.733213587
9.073536705	13.102200329	1.174773624
7.182929641	20.870139599	1.112992187
11.800479044	13.120199740	2.733213587
14.533645448	13.102200329	1.174773624
5.438978650	14.081399739	7.416583128
0.020966398	19.904399514	7.416583128
4.529725151	12.100800276	8.966599088
0.930220180	21.884999871	8.966599088
2.704993110	14.005500376	8.976520214
2.755115877	19.980300665	8.976520214
1.771660653	12.076199949	7.406005912
3.532242713	21.877795458	7.402253480
0.000000000	16.992899179	0.825083989
10.899087882	14.081399739	7.416583128
5.371272531	19.867714047	7.409234476
9.989670360	12.100800276	8.966599088

6.207301728	21.900978684	8.928424260
8.164938319	14.005500376	8.976520214
7.909304469	19.946052432	8.974544692
7.231605740	12.076199949	7.406005912
5.459945209	16.992899179	0.825083989
16.359032602	14.081399739	7.416583128
15.449779103	12.100800276	8.966599088
13.624883039	14.005500376	8.976520214
12.691551437	12.076199949	7.406005912
5.438978650	14.081399739	4.296614113
0.020966398	19.904399514	4.296614113
4.529725151	12.100800276	5.846630073
0.930220180	21.884999871	5.846630073
2.704993110	14.005500376	5.856458030
2.755115877	19.980300665	5.856458030
1.771660653	12.076199949	4.286037455
3.669248312	21.883850098	4.278371079
0.000000000	16.992899179	7.065114840
10.899087882	14.081399739	4.296614113
5.461363319	19.848235846	4.294247782
9.989670360	12.100800276	5.846630073
6.297922172	21.873114109	5.898384174
8.164938319	14.005500376	5.856458030
8.124478963	20.030313134	5.955392571
7.231605740	12.076199949	4.286037455
5.459945209	16.992899179	7.065114840
16.359032602	14.081399739	4.296614113
15.449779103	12.100800276	5.846630073
13.624883039	14.005500376	5.856458030
12.691551437	12.076199949	4.286037455
5.438978650	14.081399739	1.176645516
0.020966398	19.904399514	1.176645516
4.529725151	12.100800276	2.726567888
0.930220180	21.884999871	2.726567888
2.704993110	14.005500376	2.736489572
2.755115877	19.980300665	2.736489572
1.771660653	12.076199949	1.166068788
3.579401605	21.857569814	1.166921538
0.000000000	16.992899179	3.945146383
10.899087882	14.081399739	1.176645516
5.484984015	19.870110154	1.166813863
9.989670360	12.100800276	2.726567888
6.405997337	21.792578101	2.690286571

8.164938319	14.005500376	2.736489572
8.245530939	19.862984419	2.765687519
7.231605740	12.076199949	1.166068788
5.459945209	16.992899179	3.945146383
16.359032602	14.081399739	1.176645516
15.449779103	12.100800276	2.726567888
13.624883039	14.005500376	2.736489572
12.691551437	12.076199949	1.166068788
8.750201617	21.972704530	0.998539393
8.920391494	22.300111055	4.630525305
8.933690980	22.157185078	7.734735334

W-doped $Ni(OH)_2$
1.0

16.3799991608	0.0000000000	0.0000000000
0.0000000000	30.0000000000	0.0000000000
0.0000000000	0.0000000000	9.3599996567

Cl	H	Ni	O	W
6	71	26	66	1

Cartesian

2.730054372	16.992899179	8.687296210
8.189999580	16.992899179	8.687296210
2.730054372	16.992899179	5.567234026
8.189999580	16.992899179	5.567234026
2.730054372	16.992899179	2.447265569
8.189999580	16.992899179	2.447265569
4.541682191	11.127299666	8.959579039
0.918262773	22.858500481	8.959579039
5.439470229	15.077700019	7.394118748
0.020474998	18.908100128	7.394118748
1.746599269	11.102999747	7.397394734
3.446908332	22.834081650	7.411578212
2.712036556	15.005400181	8.942637306
2.748072432	18.980399966	8.942637306
0.793283351	16.989900470	0.230630387
10.001791423	11.127299666	8.959579039
6.458834041	22.799248695	9.047979881
10.899578973	15.077700019	7.394118748
5.424795591	18.832592368	7.420100126
7.206544478	11.102999747	7.397394734
8.171981520	15.005400181	8.942637306
8.352278303	18.974844217	8.936138336
4.666825453	16.995899677	0.230630387
6.253228499	16.989900470	0.230630387

15.461736143	11.127299666	8.959579039
16.359523693	15.077700019	7.394118748
12.666489199	11.102999747	7.397394734
13.631926241	15.005400181	8.942637306
4.541682191	11.127299666	5.839516855
0.918262773	22.858500481	5.839516855
5.439470229	15.077700019	4.274150291
0.020474998	18.908100128	4.274150291
1.746599269	11.102999747	4.277426277
3.753155064	22.837661505	4.285872038
2.712036556	15.005400181	5.822575122
2.748072432	18.980399966	5.822575122
0.793283351	16.989900470	6.470661255
10.001791423	11.127299666	5.839516855
6.321910942	22.774466872	5.607728042
10.899578973	15.077700019	4.274150291
5.530390405	18.632238507	4.276312988
7.206544478	11.102999747	4.277426277
8.171981520	15.005400181	5.822575122
4.666825453	16.995899677	6.470661255
6.253228499	16.989900470	6.470661255
15.461736143	11.127299666	5.839516855
16.359523693	15.077700019	4.274150291
12.666489199	11.102999747	4.277426277
13.631926241	15.005400181	5.822575122
4.541682191	11.127299666	2.719547840
0.918262773	22.858500481	2.719547840
5.439470229	15.077700019	1.154181555
0.020474998	18.908100128	1.154181555
1.746599269	11.102999747	1.157363953
3.719764782	22.886738777	1.235151734
2.712036556	15.005400181	2.702606386
2.748072432	18.980399966	2.702606386
0.793283351	16.989900470	3.350599071
10.001791423	11.127299666	2.719547840
6.622750059	22.770325541	2.953293485
10.899578973	15.077700019	1.154181555
5.470104349	18.839875460	1.191822752
7.206544478	11.102999747	1.157363953
8.171981520	15.005400181	2.702606386
8.316591707	18.753851652	2.686121294
4.666825453	16.995899677	3.350599071
6.253228499	16.989900470	3.350599071

15.461736143	11.127299666	2.719547840
16.359523693	15.077700019	1.154181555
12.666489199	11.102999747	1.157363953
13.631926241	15.005400181	2.702606386
9.772521250	21.725395918	1.703357833
9.246083427	21.065801382	0.358163988
9.524514443	21.944906116	5.429140521
10.038189759	21.206556559	4.133027950
9.868490974	22.447822094	7.914592076
9.666158558	20.971793532	7.470674269
0.880424970	13.120199740	8.973151059
4.557161809	20.845402479	9.033708813
3.613591496	13.102200329	7.414804544
1.846353469	20.883600712	7.414804544
6.340370300	13.120199740	8.973151059
9.073536705	13.102200329	7.414804544
7.173285024	20.742684603	7.548151440
11.800479044	13.120199740	8.973151059
14.533645448	13.102200329	7.414804544
0.880424970	13.120199740	5.853182044
4.527555271	20.835562348	5.909312866
3.613591496	13.102200329	4.294835808
1.846353469	20.883600712	4.294835808
6.340370300	13.120199740	5.853182044
9.073536705	13.102200329	4.294835808
11.800479044	13.120199740	5.853182044
14.533645448	13.102200329	4.294835808
0.880424970	13.120199740	2.733213587
4.562518411	20.832771063	2.742943632
3.613591496	13.102200329	1.174773624
1.846353469	20.883600712	1.174773624
6.340370300	13.120199740	2.733213587
9.073536705	13.102200329	1.174773624
7.287536903	20.754160881	1.204901868
11.800479044	13.120199740	2.733213587
14.533645448	13.102200329	1.174773624
5.438978650	14.081399739	7.416583128
0.020966398	19.904399514	7.416583128
4.529725151	12.100800276	8.966599088
0.930220180	21.884999871	8.966599088
2.704993110	14.005500376	8.976520214
2.755115877	19.980300665	8.976520214
1.771660653	12.076199949	7.406005912

3.564752595	21.868141294	7.435753669
0.000000000	16.992899179	0.825083989
10.899087882	14.081399739	7.416583128
5.403476091	19.834715724	7.460884250
9.989670360	12.100800276	8.966599088
6.391135733	21.830611825	9.051898009
8.164938319	14.005500376	8.976520214
8.277329812	19.975633621	8.975605817
7.231605740	12.076199949	7.406005912
5.459945209	16.992899179	0.825083989
16.359032602	14.081399739	7.416583128
15.449779103	12.100800276	8.966599088
13.624883039	14.005500376	8.976520214
12.691551437	12.076199949	7.406005912
5.438978650	14.081399739	4.296614113
0.020966398	19.904399514	4.296614113
4.529725151	12.100800276	5.846630073
0.930220180	21.884999871	5.846630073
2.704993110	14.005500376	5.856458030
2.755115877	19.980300665	5.856458030
1.771660653	12.076199949	4.286037455
3.784116251	21.866875291	4.304841177
0.000000000	16.992899179	7.065114840
10.899087882	14.081399739	4.296614113
5.619335477	19.656284451	4.349590850
9.989670360	12.100800276	5.846630073
6.366114989	21.824313998	5.802535931
8.164938319	14.005500376	5.856458030
8.051609554	19.898843765	5.941006576
7.231605740	12.076199949	4.286037455
5.459945209	16.992899179	7.065114840
16.359032602	14.081399739	4.296614113
15.449779103	12.100800276	5.846630073
13.624883039	14.005500376	5.856458030
12.691551437	12.076199949	4.286037455
5.438978650	14.081399739	1.176645516
0.020966398	19.904399514	1.176645516
4.529725151	12.100800276	2.726567888
0.930220180	21.884999871	2.726567888
2.704993110	14.005500376	2.736489572
2.755115877	19.980300665	2.736489572
1.771660653	12.076199949	1.166068788
3.743132121	21.916115284	1.219655380

0.000000000	16.992899179	3.945146383
10.899087882	14.081399739	1.176645516
5.487078718	19.838631749	1.239116586
9.989670360	12.100800276	2.726567888
6.585287042	21.801338196	2.947078206
8.164938319	14.005500376	2.736489572
8.266985659	19.787104726	2.849795325
7.231605740	12.076199949	1.166068788
5.459945209	16.992899179	3.945146383
16.359032602	14.081399739	1.176645516
15.449779103	12.100800276	2.726567888
13.624883039	14.005500376	2.736489572
12.691551437	12.076199949	1.166068788
9.236192288	21.917104125	0.912816189
9.311894501	21.778619885	4.458591014
9.628535911	21.897681355	7.145481372
7.405589123	20.513037443	4.430063104

Supplementary References

1 P. Zhang, L. Li, D. Nordlund, H. Chen, L. Fan, B. Zhang, X. Sheng, Q. Daniel, L. Sun, *Nature Commun.* **2018**, *9*, 381.

2 H. Liang, A. N. Gandi, C, Xia, M. N. Hedhili, D. H. Anjum, U. Schwingenschlögl, H. N. Alshareef, *ACS Energy Lett.* **2017**, *2*, 1035-1042.

3 J. Zhang, J. Liu, L. Xi, Y. Yu, N. Chen, S. Sun, W. Wang, K. M. Lange, B. Zhang, *J. Am. Chem. Soc.* **2018**, *140*, 3876-3879.

4 P. Li, X. Duan, Y. Kuang, Y. Li, G. Zhang, W. Liu, X. Sun, *Adv. Energy Mater.* **2018**, *8*, 1703341.

5 K. Fan, H. Chen, Y. Ji, H. Huang, P. M. Claesson, Q. Daniel, B. Philippe, H. Rensmo, F. Li, Y. Luo, L. Sun, *Nature Commun.* **2016**, *7*, 11981.

6 H. Liang, F. Meng, M. Cabán-Acevedo, L. Li, A. Forticaux, L. Xiu, Z. Wang, S. Jin, *Nano Lett.* **2015**, *15*, 1421-1427.

7 Y. Yang, L. Dang, M. J. Shearer, H. Sheng, W. Li, J. Chen, P. Xiao, Y. Zhang, R. J. Hamers, S. Jin, *Adv. Energy Mater.* **2018**, *8*, 1703189

8 H. Zhang, X. Li, A. Hähnel, V. Naumann, C. Lin, S. Azimi, S. L. Schweizer, A. W. Maijenburg, R. B. Wehrspohn, *Adv. Funct. Mater.* **2018**, *28*, 1706847.

9 X. Long, J. Li, S. Xiao, K. Yan, Z. Wang, H. Chen, S. Yang, *Angew. Chem. Int. Ed.* **2014**, *53*, 7584-7588.

10 W. Ma, R. Ma, C. Wang, J. Liang, X. Liu, K. Zhou, T. Sasaki, *ACS Nano* **2015**, *9*, 1977-1984.

11 X. Kong, C. Zhang, S. Y. Hwang, Q. Chen, Z. Peng, *Small* **2017**, *13*, 1700334.

12 G. Chen, T. Wang, J. Zhang, P. Liu, H. Sun, X. Zhuang, M. Chen, X. Feng, *Adv. Mater.* **2018**, 1706279.

13 M. Asnavandi, Y. Yin, Y. Li, C. Sun, C. Zhao, *ACS Energy Lett.* **2018**, *3*, 1515-1520.

14 J. Jiang, F. Sun, S. Zhou, W. Hu, H. Zhang, J. Dong, Z. Jiang, J. Zhao, J. Li, W. Yan, M. Wang, *Nature Commun.* **2018**, *9*, 2885.
